# A matter of time: A systematic scoping review on a potential role of the circadian system in binge eating behavior

**DOI:** 10.3389/fnut.2022.978412

**Published:** 2022-09-08

**Authors:** Francisco Romo-Nava, Anna I. Guerdjikova, Nicole N. Mori, Frank A. J. L. Scheer, Helen J. Burgess, Robert K. McNamara, Jeffrey A. Welge, Carlos M. Grilo, Susan L. McElroy

**Affiliations:** ^1^Lindner Center of HOPE, Mason, OH, United States; ^2^Department of Psychiatry and Behavioral Neuroscience, University of Cincinnati College of Medicine, Cincinnati, OH, United States; ^3^Medical Chronobiology Program, Division of Sleep and Circadian Disorders, Department of Medicine and Neurology, Brigham and Women’s Hospital, Boston, MA, United States; ^4^Division of Sleep Medicine, Harvard Medical School, Boston, MA, United States; ^5^Sleep and Circadian Research Laboratory, Department of Psychiatry, University of Michigan, Ann Arbor, MI, United States; ^6^Department of Environmental and Public Health Sciences, University of Cincinnati College of Medicine, Cincinnati, OH, United States; ^7^Department of Psychiatry, Yale School of Medicine, New Haven, CT, United States

**Keywords:** binge eating, circadian, chronobiology, actigraphy, obesity, chronotype, light, night eating

## Abstract

**Background:**

Emerging research suggests that food intake timing, eating behavior and food preference are associated with aspects of the circadian system function but the role that the circadian system may play in binge eating (BE) behavior in humans remains unclear.

**Objective:**

To systematically evaluate the evidence for circadian system involvement in BE behavior.

**Methods:**

Systematic searches of PubMed, EMBASE, and Scopus were performed for reports published from inception until May 2020 (PROSPERO Registration CRD42020186325). Searches were conducted by combining Medical Subject Headings related to the circadian system, BE behavior, and/or interventions. Observational and interventional studies in humans with BE behavior published in peer-review journals in the English language were included. Studies were assessed using quality and risk of bias tools (AXIS, ROB 2.0, or ROBINS).

**Results:**

The search produced 660 articles, 51 of which were included in this review. Of these articles, 46 were observational studies and 5 were interventional trials. Evidence from these studies suggests that individuals with BE behavior tend to have more food intake, more binge cravings, and more BE episodes later in the day. Hormonal and day/night locomotor activity rhythm disturbances may be associated with BE behavior. Furthermore, late diurnal preference (“eveningness”) was associated with BE behavior and chronobiological interventions that shift the circadian clock earlier (e.g., morning bright light therapy) were found to possibly decrease BE behavior. Substantive clinical overlap exists between BE and night eating behavior. However, there is a significant knowledge gap regarding their potential relationship with the circadian system. Limitations include the lack of studies that use best-established techniques to assess the chronobiology of BE behavior, heterogeneity of participants, diagnostic criteria, and study design, which preclude a meta-analytic approach.

**Conclusion:**

Current evidence, although limited, suggests that the circadian system may play a role in the etiology of BE behavior. Further mechanistic studies are needed to fully characterize a potential role of the circadian system in BE behavior. A chronobiological approach to studying BE behavior may lead to identification of its neurobiological components and development of novel therapeutic interventions.

**Systematic review registration:**

[https://www.crd.york.ac.uk/prospero/display_record.php?ID=CRD42020186325], identifier [CRD42020186325].

## Introduction

Binge eating (BE) behavior is a form of disordered eating characterized by consuming an objectively large amount of food in a short amount of time with a sense of loss of control over eating (1). BE behavior is a core feature of the eating disorders (EDs) binge-eating disorder (BED) and bulimia nervosa (BN), and may occur in anorexia nervosa (AN). BE behavior may also occur in night eating syndrome (NES), classified as an Other Specified Feeding or Eating Disorder in the *DSM-5*, as well as in non-ED-psychiatric diagnoses like mood and attention-deficit/hyperactivity disorder (ADHD) (2–5). Currently, BE behavior is often identified as a symptom associated with deficits in impulse-control but its neurobiology remains poorly understood (3).

The circadian system is a robust multi-oscillator circadian network influencing most physiological and behavioral processes, including metabolism, hunger, food intake timing, and eating behavior (6–8). The circadian system consists of the suprachiasmatic nucleus (also called the master pacemaker) located in the hypothalamus and of peripheral oscillators or “clocks” located in other regions of the brain and most tissues of the body (8, 9). Inadequate or mistimed interactions among components of the circadian system with the environmental and/or behavioral cycle can have profound physiological consequences and are associated with multiple adverse health outcomes, which may include disordered eating (9–14). Early studies suggest that time-of-day clinical features are associated with BE behavior (15, 16) and that targeted chronobiological interventions may have therapeutic potential in EDs (17), suggesting a possible circadian system involvement in BE behavior not yet thoroughly explored. Moreover, variability across the day and night in a physiological variable can be the result of changes in behavior or environmental conditions cycling in parallel and do not necessarily reflect involvement of the circadian system.

Best-established techniques like constant routine (CR) or forced desynchrony (FD) are typically used to discern a change in the circadian rhythm in physiology and behavior by minimizing the influence of behavioral and environmental effects or by evenly distributing them during the circadian cycle (18). However, these techniques are typically conducted in well-controlled experimental conditions and have not been applied to study the role of the circadian system in BE behavior.

Emerging research supports that food intake timing (10, 19), eating behavior and food preference (12, 14, 20) are associated with aspects of the circadian system function in humans but the role that the circadian system may play in BE behavior remains unclear. Given the absence of published studies using best-established methods to study the circadian system in BE behavior, we aimed to systematically review current evidence on circadian-related or proxy measures that can inform the potential role of the circadian system in the etiology, phenomenology, and treatment of BE behavior.

## Method

Initially, a systematic review and meta-analysis approach was conducted following the Preferred Reporting Items for Systematic Reviews and Meta-Analyses (PRISMA) checklist guidelines (Supplementary Table 1) (21). The study protocol was registered with the International Prospective Register of Systematic Reviews (PROSPERO); registration number CRD42020186325. The limitations on available evidence precluded a quantitative analysis on each of the specific sub-categories initially planned to conduct the systematic review and meta-analysis approach. In this context, the complex and heterogeneous nature of the current body of evidence gave way to a systematic scoping review on the existing literature in this topic (22).

## Database searches

Searches for relevant scientific articles were conducted in PubMed, EMBASE, and Scopus from inception to May 1st, 2020. Observational and interventional studies in humans published in peer-review journals in the English language were included. References from reviews, systematic reviews, and meta-analyses were also evaluated to identify additional original studies.

Observational articles included were those reporting on human BE behavior and some aspect of a circadian-related function. Studies included individuals with BE behavior and/or a diagnosis of BED, BN, AN, or NES. We included night eating because of its clinical overlap with BE behavior (23, 24). Aspects of circadian-related function included: (1) timing of BE behavior; (2) timing of food intake; (3) a behavioral assessment of chronotype (e.g., dimensional or categorical); or (4) objective evaluations of a circadian-related function (e.g., dim light melatonin onset, actigraphy or sleep/wake cycle).

Interventional trials included were those evaluating chronotherapeutic strategies in individuals with BE behavior and reporting on BE behavior as an outcome. Interventions of chronobiological relevance included light therapy, melatonergic agents, or scheduled food intake. Other interventions that may influence the circadian system via changes in sleep/wake cycles or the timing of light exposure targeting the circadian system in conditions with BE behavior (e.g., interpersonal and social rhythms therapy) were considered if their effect on a circadian-related assessment (e.g., circadian phase, actigraphy) was reported.

Specific search terms used for circadian-related factors were: suprachiasmatic nuclei or nucleus, biological clock, circadian, chronotype, morningness-eveningness, morning, evening, night eating, nocturnal binge, and skipping meals (e.g., breakfast). The following specific search terms for BE behavior and food intake timing were used: anorexia nervosa, bulimia nervosa, night eating, night eating syndrome, BE, and food intake timing or food intake time (or temporal pattern). Specific terms for chronological interventions were the following: bright light, light therapy, melatonin, melatonin agonist, melatonergic, and scheduled food intake.

## Data extraction

All potentially relevant studies were screened by two authors (FR-N, AG, or NM) after independent evaluation of titles and abstracts. A third author (SM, FR-N, AG, or NM) made a final decision when there was disagreement between reviewers regarding the eligibility of a study. Upon completion of the screening process, a list of studies was compiled and reviewed for repetitions. Full articles were then reviewed to assess eligibility. Additional studies were selected when identified after reviewing a study. Two authors discussed the findings (FR-N, AG, or NM) and a third author (FR-N, AG, NM, or SM) helped make a final decision when there was disagreement. Reviewer agreement was substantial (Fleiss’ kappa = 0.72). A critical appraisal of selected observational studies was conducted using the Appraisal tool for Cross-Sectional Studies (AXIS tool) (25) and is presented separately for each section (Supplementary Tables 2–4). For the interventional studies risk of bias was assessed using the COCHRANE ROBINS for non-randomized trials or Rob 2.0 tools for randomized trials (see Supplementary Figures 1, 2, respectively) (26, 27). The PRISMA flowchart was followed for two categories of studies: observational and interventional. A data extraction form developed by the authors was used to systematically collect data from identified studies.

## Strategy for data synthesis

Although considered initially, a meta-analytic approach for data synthesis was not possible due to the small number of studies meeting criteria for each subsection and their considerable methodological differences (heterogeneity). We therefore classified the 51 identified studies into two categories: observational and interventional, and conducted narrative syntheses of each category. Potential links between the categories and sections were addressed in the discussion.

## Results

### Observational studies

**FIGURE 1:**
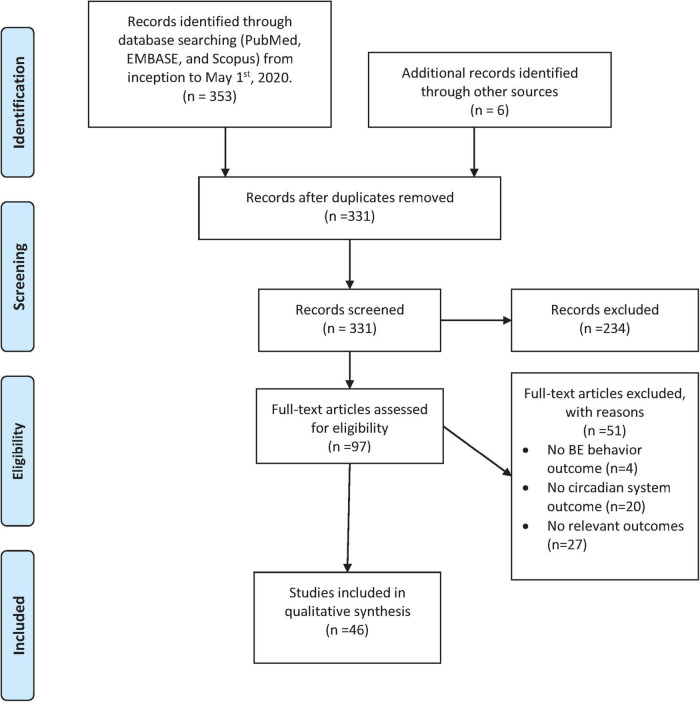
PRISMA flowchart for observational studies.

Forty-six observational studies reported relevant information on circadian-related features of BE behavior and/or food intake patterns, circadian-related outcome measures, or night eating among individuals with BE behavior (see Figure 1). Because these studies had heterogeneous methods, we elected to summarize them in three main sections: (1) patterns of food intake and/or timing of BE behavior (*N* = 16); (2) assessment of the circadian-related measures among individuals with BE behavior (*N* = 12); and (3) the co-occurrence of BE behavior and night eating (*N* = 18).

#### Patterns of food intake and/or timing of binge eating behavior

Sixteen studies reported information on time-of-day related features of BE behavior and/or food intake among individuals with BE behavior (Table 1 and Supplementary Table 2). Nine of these studies explored food intake patterns throughout the day among individuals with BE behavior (15, 28–35); five explored timing of BE behavior or of the urge to binge eat (16, 36–39); one study evaluated the relationship between BE behavior and photoperiod (40); and one study evaluated food intake patterns in youth with BE behavior (41).

**TABLE 1:** Studies on patterns of food intake and/or timing of BE behavior.

Author, year	Study design	Participants and age (*SD*)	Sample size/Female%	Outcomes	Instruments	Results	Comments and limitations
Blouin et al. (40)	Study 1: CS Study 2: CS	NW women with BN vs. Ctrl Study 1: BN = 24.6(6.0), Ctrl = 24.7(6.2) Study 2: BN = 26	Study 1: BN = 31, Ctrl = 31 F = 100% Study 2: BN = 197/F = 98%	Seasonal patterns in BN (DSM III-R) Association between BE and photoperiod	Study 1: M-SPAQ Study 2: Self-report questionnaire	Study 1: Dark hours predicted% of subjects with BE likelihood each month. SAD in 35.5% of BN. Study 2: BE/week directly correlated with dark hours in the month of assessment in all subjects. Purging did not show this correlation	Dark hours included twilight hours at dusk and dawn. Retrospective and self-reported assessments. BE time of day not assessed. High% with psychiatric comorbidities
Cachelin et al. (35)	CS	Latina women with/o ED All = 26.9 (?)	BED = 65 BN = 22 No ED = 68/F = 100%	Eating patterns in Latina women with BE	*DSM-5* criteria	BN had more nocturnal eating than the other two groups (BN: 18.2%; BED: 3.1%; no ED: 1.5%). BED (48%) and BN (68%) snacked more frequently during the evening (more than half of the past 28 days). In BED, BN and Ctrl group no association between breakfast or evening snacks and BE	Association between meal timing and BE was not analyzed. BE timing was not assessed
Ellison et al. (34)	P	BN or partial BN (purging + objective or subjective BE) All = 27.3 (9.6)	BN = 68/F = 90%	Association between evening meals and BE	EDE	Increased evening meal consumption during treatment predicted decreased BE after 4-month follow-up. Similar results with depression as covariate.	Subjects were enrolled in a RCT and receiving psychotherapy focusing on food.
Elran-Barak et al. (41)	CS	Children and adolescents with AN-R = 14.7(2.1) AN BE/P = 16.26(1.9)	AN-R = 120 AN-BE/P = 40 F = 93.8%	Meal patterns in youth with AN types	*DSM-5* criteria EDE	Youth with AN-BE/P consumed less breakfast, lunch, dinner, as well as mid-morning and mid-afternoon snacks compared to AN-R types. BE-P type that consumed dinner more often had less BE episodes.	
Ferrer-Garcia et al. (38)	CS	BED and BN patients All = 30.1 (8.02)	Total = 101 BED = 50, BN = 51/F = 88%	Transcultural contexts and cues that elicit food craving	*DSM-5* criteria	BE craving was higher in the afternoon/early evening and late evening/night.	
Harvey et al. (31)	CS	Women with BED or RBE (1/week for 3 months) All = 33.9(7.4)	Total = 106 BED = 48, RBE = 58 F = 100%	Meal patterns in BED and RBE women	*DSM-IV* criteria EDE	Dinner was the most and breakfast the least common meal. Evening snacking was most frequent and associated with BE. Meal frequency not associated with BE, BMI, or ED pathology	BE timing not assessed. Correlations for meal patterns and BE was not provided separately for BED and RBE
Leblanc et al. (32)	P (3-days)	Premenopausal women with overweight or OB All = 42.6 (5.6)	Total = 143/F = 100%	Association between eating behavior and eating patterns	TFEQ Self-report dietary intake	BES scores positively correlated with% energy intake after 5 pm. This correlation was not significant after removal of “underreporters.” % of energy intake from snacks after 5 pm mediates the association between BES scores and self-reported energy intake	BE frequency and timing not assessed. Presence of ED in participants not reported. Self report
Masheb et al. (30)	CS	BED, BN, and Ctrl Community volunteers All = 36.3 (12.4)	Total = 311 BED = 69, BN = 39, Ctrl = 203/F = 100%	Eating behavior and patterns in BED or BN, and controls	EDE-Q	BED > snacks/day and evening snacks than controls, but not compared to BN. BED and BN > nocturnal eating compared to controls More frequent breakfast related to < weight in BED and control, but not BN. In BED, BE inversely related to dinner frequency.	BE timing not assessed Dx based on self-report
Masheb and Grilo (29)	CS	BED All = 45.2 (8.8)	Total = 173/F = 73%	Meal patterns in BED	SCID-I/P (*DSM-IV*), EDE, TFEQ	No association between meal frequency and BE Evening snack directly correlated with BE days and episodes	BE timing not assessed
Mitchell et al. (36)	CS	BN All = 24.8 (?)	Total = 275/F = 100%	Description of clinical variables in BN	*DSM-III*	Subjects that reported BE usually occurring late afternoon (46.2%), early evening (52.4%), late evening (54.2%), or after midnight (15.3%). BE was reported as usual early in the morning (17.1%) or late morning (17.5%). BE could occur at any time in (33.5%)	BMI not reported
Pla-Sanjuanelo et al. (39)	CS	BED and BN = 30.1 (8.0) Ctrl = 22.64 (6.06)	Total = 101 BED = 50, BN = 51/F = 88% Ctrls = 63/F = 85%	Binge craving and specific cues	*DSM-5* EDE-3 BULIT-R PBEBI	Higher binge craving at dinner, snacking between meals (after dinner), during the afternoon/evening, and between 8 and 12 pm	
Raymond et al. (15)	P	Obese women with BED BED = 37.9(7.8) Control = 34.9(8.0)	BED = 12 Control = 8/F = 100%	Energy intake patterns in obese women with BED	SCID-IIIR *DSM-IV* BED criteria EDE, Dietary recall interviews	DEC in BED = Ctrl group. BED group had a higher calory intake (50% of DEC) in the evening compared to Ctrl (39% of DEC). In BE days, BED consumed less calories in the morning and midday compared to Ctrl. Similar patterns of energy intake between BE days and No-BE days.	BE timing not assessed Small sample size
Shah et al. (33)	P	Women with BN BN = 28.7(7.2)	BN = 158/F = 100%	Association between eating patterns and BE	EDE	Highest BE behavior abstinence rates in those with more meals and more afternoon or less evening snacks.	Participants received CBT or IBT during study follow-up.
Schreiber-Gregory et al. (16)	Study 1: CS Study 2: P	BED subjects Study 1 = 47.5(10.8) Study 2 = 37.9(11.8)	Study 1: BED = 139/F = 87.7% Study 2: BED = 5 Subthreshold BED = 4/F = 100%	BE duration and temporal pattern in BED	Study 1:EDE Study 2: SCID-IV E-recording of BE and meals	Study 2: BE more frequent in the early afternoon (12–3 pm) and evening hours (6–9 pm) BE less frequent during the weekend days	Study 1: BE timing not assessed Study 2: Small sample size, included subthreshold BED
Waters et al. (37)	P (1-week)	Women with BN All = 24.8 (SE 0.7)	Total = 15/F = 100%	Factors preceding BE	BE diary and craving record	Time of day had an effect on BE after craving BE more frequent in the afternoon	Statistical model underpowered
Weltzin et al. (28)	P	Women BN BN = 24.8(6.3) Ctrl = 22.5(4.0)	BN = 54 Ctrl = 11/F = 100%	Food intake patterns in BN	SCID-*DSM-III* criteria. Conducted in laboratory conditions	Total calories BN > Controls. # meals/day BN = Controls BN: majority of meals and calories between noon and midnight	Study conducted in laboratory conditions

ADHD-RS, attention deficit and hyperactivity disorder-rating scale; AN, anorexia nervosa; AN-B/P, anorexia nervosa-bingeing/purging type; AN-R, anorexia nervosa-restrictive type; BE, binge eating; BED, binge eating disorder; BES, binge eating scale; BMI, body mass index; BN, bulimia Nervosa; BULIT-R, bulimia test-revised; CBT, cognitive behavioral therapy; CS, cross-sectional; Ctrl, control; DEC, daily energy consumption; DSPS, delayed sleep phase syndrome; Dx, diagnosis; ED, eating disorder; EDE, eating disorder questionnaire; EDI-3, eating disorder inventory-3; F, female; IPT, interpersonal therapy; M-SPAQ, Modified (Binge-Purging) Seasonal Pattern Assessment Questionnaire; NW, normal weight; OB, obesity; PBEBI, precipitating binge eating behavior inventory; P, prospective; RBE, recurrent binge eating; RCT, randomized controlled trials; SCID III, structured clinical diagnostic interview for the DSM-III; SD, standard deviation; TFEQ, Three Factor Eating Questionnaire; VOA, Dutch version of a Morning/Evening type Questionnaire.

##### Daily food intake patterns

The first of nine studies exploring daily food intake patterns were conducted under laboratory conditions across a 24-h period (divided in four 6-h periods) in female patients with BN (*n* = 54) and showed a disturbed eating pattern characterized by the absence of the typical peaks in meal intake patterns during breakfast, lunch, and dinner that were observed in the control group (*n* = 11). In addition, 30% of the meals consumed between the afternoon (18% from 12 to 6 pm) and midnight (12% from 6 pm to 12 am) periods were considered “large” (i.e., a > 1000 kcal meal) in a subset of BN patients (*n* = 24) cataloged as “overeaters,” with a 24-h caloric intake of more than two standard deviations higher than the mean of the control group (28). The combined percentage of meals that were considered “large” from midnight to noon was only 8%. Unfortunately, it was not reported whether the overeating episodes met criteria for BE behavior. In the second study, calorie intake in women with BED (*DSM-IV* criteria) and obesity was lower in the mornings and midday, and higher in the evening, when compared to a BMI and age matched control group with obesity without BE behavior (15). The third study found that among 173 male and female patients with BED (*DSM-IV* criteria) with co-existing obesity, the frequency of evening snacking was directly correlated with the frequency of BE behavior days and BE behavior episodes per week (29). This correlation was not observed with the frequency of other meals individually or with the overall meal frequency. In the fourth study, eating patterns were evaluated among women with BN (*n* = 39) and BED (*n* = 69) defined by modified-*DSM*-IV criteria from a community sample (30). It reported that BE behavior frequency was inversely correlated with the average number of meals per day and with dinner frequency only in the BED group. This was interpreted as indicating that BED individuals with less regular meals and those who consumed dinner less frequently experienced more BE behavior episodes. In the same study, more frequent breakfast consumption was associated with lower weight in the BED group.

In the fifth study, 106 individuals with *DSM*-IV BED or recurrent BE behavior (e.g., one episode/week for at least 3 months, similar to current *DSM*-5 BED criteria) reported that dinner and evening snacks were the most common meal and snacks consumed, respectively, and that the number of days with BE behavior was directly correlated with the frequency of evening snacks (31). The sixth study found that severity of BE behavior correlated directly with the proportion of energy intake from snacks after 5:00 pm among premenopausal women with overweight or obesity (32). Of note, a secondary analysis identified that the association between BE behavior severity and total self-reported energy intake was partially mediated by the percentage of energy intake from evening snacks after 5:00 pm. In the seventh study, a prospective evaluation of 158 patients with BN, an increase in regular meals and more afternoon or evening snacks were both associated with increased abstinence rates of BE and purging behavior (33). In the eight study, also using a prospective design, increased evening meal intake frequency was associated with a decrease in BE behavior frequency in 68 patients with BN or partial BN (purging + objective or subjective BE behavior) (34). The ninth study showed no correlation between meal intake frequency and BE behavior frequency among Latina women with BN (*n* = 22) or BED (*n* = 65) (35). A limitation in these studies was that BE behavior timing and its relationship with meal and snack timing was not analyzed. Nonetheless, taken together, these studies suggest more regular meal and snack intake during the later part of the day among individuals with BE behavior is associated with less BE behavior.

##### Binge eating behavior timing

Five studies examined BE behavior timing. In the first study, with 275 patients with BN, the early evening (52.4%) and late evening (54.2%) were reported as the time of day when BE behavior usually occurred for more than half of participants (36). The second study was a one-week prospective investigation of the internal and external factors that preceded BE behavior occurrence after an urge to binge eat in 15 women with BN (37). This study identified that both the urge to binge eat and BE behavior occurred more frequently during the afternoon. An effect of time of day of the urge to binge eat on BE behavior was also reported. However, the statistical model was underpowered and results difficult to interpret. The third study utilized an ecological moment assessment approach to prospectively evaluate the duration and timing of BE behavior episodes in nine subjects with threshold (*n* = 5) and subthreshold BED (*n* = 4) according to *DSM-IV* criteria (16). Frequency of BE behavior was higher in the early afternoon (12:00 to 3:00 pm) and evening (6:00 to 9:00 pm) than in the morning. Interestingly, BE behavior was less frequent during weekend days than on weekdays. The fourth and fifth studies reported only on the timing of the urge (or craving) to binge eat in subjects with BN or BED. In both studies, the urge to binge eat was heightened in the afternoon/early evening (4:00 pm to 8:00 pm), late evening/night (8:00 pm to 12:00 am), and was consistently increased during the weekend (38, 39). These observations further suggest that the more frequent occurrence of BE behavior during the evening or night is not just explained by an “increased opportunity” to binge eat when individuals are not busy or working (e.g., after work hours) even if the craving to binge eat is high during the weekends.

##### Photoperiod and binge eating behavior

One study evaluated the relationship between BE behavior and photoperiod, defined as the average number of light hours across 24-h. It was observed that among 31 patients with BN, a decreased number of light hours predicted an increased likelihood for BE behavior each month but did not predict the likelihood of purging behavior (40). It was concluded that photoperiod may exert an influence over BE behavior that is likely mediated by the circadian system and is independent from other compensatory behaviors (e.g., self-induced vomiting, misuse of laxatives, weight loss medications, or diuretics, food restriction or prolonged fasting, or excessive exercise), although—as recognized by the authors—a role independent of other highly correlated seasonal changes such as environmental temperature could not be determined.

##### Patterns of food intake in children and adolescents with binge eating behavior

One study explored food intake patterns in youth with BE behavior. This study evaluated 160 children and adolescents with restrictive AN (AN-R, *n* = 120) or binge eating/purgative AN (AN BE/P, *n* = 40) subtypes. Youth with AN-BE/P consumed breakfast, lunch, dinner, as well as mid-morning and mid-afternoon snacks less often as compared to those with AN-R types. There were no group differences in evening snack consumption. Interestingly, as has been described in adults with BE behavior, youth with AN BE/P type with a less regular dinner consumption experienced more BE behavior episodes (41).

#### Assessment of circadian-related measures among individuals with binge eating behavior

Twelve studies provided evidence on objective or subjective circadian-related parameters in individuals with BE behavior (see Table 2 and Supplementary Table 3). Assessment methods used to explore circadian-related function in these studies included hormonal levels (*n* = 3), actigraphy (*n* = 5), diurnal preference or chronotype (*n* = 3), and genetics (*n* = 1). These studies are summarized below.

**TABLE 2:** Assessment of circadian-related measures among individuals with BE behavior.

Author, year	Study design	Participants, Age	Sample size/Female%	Outcomes	Instruments	Results	Limitations and comments
Carnell et al. (42)	CS	Obesity w/and w/o BED BED = 35.9(8.1), OB = 36.8(9.2)	*N* = 32, BED = 16(62.5%), OB = 16(56.3%)	Time of day and between group differences in hunger/fullness, food intake, and ghrelin levels	IDS, PSS, TEFQ, DEBQ, ZUNG, EMAQ, PFS, BES, STAI, NESHI	PM eating associated w/↑ hunger and ↓ fullness in BED. Fasting ghrelin ↓ in AM and ↑ in PM in BED. Greater experience of LOC and BE resemblance at AM and PM in BED	Limited information on BE behavior frequency/severity for BED participants.
Galasso et al. (46)	P	BED with obesity, 56.8(16.7) Controls with obesity = 61.5(13.8)	*N* = 28(100%), BED with obesity = 14, Controls with obesity = 14	Relationship between RAR and BED diagnosis	Wrist actigraphy	BED showed ↓ MESOR and amplitude and poor sleep quality compared to controls. No difference in acrophase.	BED actigraphy recordings while on intensive cognitive-behavioral therapy and nutritional program (8–5 p.m). Controls did not receive intervention
Harb et al. (49)	CS	Patients with obesity seeking weight loss treatment 39.5(11.7)	*N* = 100 (77%)	Associations between chronotype, eating patterns and BMI	MEQ, NEQ, EAT	Strong association between eveningness and BE, weak association w/number of night eating behaviors	Low incidence of night eating
Mason et al. (47)	P	DSM-5 BN, DSM-5 BED 44.28 (12.54)	*N* = 212(85%)	Sleep disturbance in obesity. Association between ED severity and subjective sleep measures	13-CSM (used in place of MEQ), EDDS, SATED	BN and BED associated w/↑eveningness, ↓subjective sleep. ↑ED severity correlated with ↓ sleep quality	Patients seeking weight loss treatment, self-report measures
Monteleone et al. (52)	CS	Obesity w/and w/o BED. Age reported by Genotype	*N* = 298 (85.4%), OB = 99, OB-BED = 107	CLOCK polymorphism in obesity. Eveningness in homozygous T111C genotype	SCID-IP	3111T/C associated w/↑BMI in obesity, not w/BED	No circadian-related measures. Ctrls significantly younger.
Romo-Nava et al. (12)	CS	Non-evening BD = 40.8 (14.8); Evening type BD = 37.5 (13.4)	*N* = 783; non-evening = 575(66%) Evening = 208(60%)	Association between chronotype and unhealthy eating behaviors	EDDS, REAPS	Evening chronotype associated w/increased eating psychopathology (EDDS scores), higher BE behavior, BN, and nocturnal eating binges, and BMI. Evening types had worse dietary habits (REAPS scores), including skipping breakfast more often, eating less fruits and vegetables, consumed more fried foods and unhealthy snacks.	Retrospective survey. No control for effects of medications or BMI.
Roveda et al. (48)	P	OB with BED = 55.7(15.6), OB w/o BED = 60.0(12.4)	*N* = 16(100%), OB with BED = 8, OB w/o BED = 8	Relationship between RAR and BED diagnosis	Actigraphy	BED showed ↓ MESOR and amplitude and poor sleep quality compared to controls. No difference in acrophase or sleep quality.	BED participant actigraphy recorded during day hospital care with a “multidisciplinary treatment”
Taylor et al. (43)	P	NW females w/normal menstrual cycles = 28.1(3.0)	*N* = 7(100%)	Effects of BE-like dinner on metabolism	BITE (Edinburgh Bulimic investigation Test)	Time of eating drove diurnal leptin rhythm. BE-like dinner increased fasting glucose levels, and increased postprandial insulin without a change in postprandial glucose levels	Small sample size. Metabolic parameters measured only during 14 h. Participants fasted prior to the BE-like dinner
Tzischinsky et al. (44)	P	Obesity with BED = 37.8(5.5), Obesity w/o BED = 38.0(6.7) NW = 22.1(3.1)	*N* = 47(100%), Obesity with BED = 18, Obesity w/o BED = 13, NW = 16	Characterization of sleep disturbance in BED and OB	1-week Actigraphy, MSQ, Std Technion Clinical Sleep Questionnaire, Sleep diary	Compared to NC, the BED and OB group showed sleep disturbance, ↓quality, and on actigraphic measures for SE, TST, Long, Zero, and WAKE	No assessment of mediators in sleep disturbance (e.g., OSA)
Tzinchinsky and Latzer (45)	P	Children with OB with and w/o BE. OB with BE = 9.7(2.0), OB w/o BE = 9.1(1.70), NW = 10.2(1.5),	*N* = 61, OB-BE = 13 (38.5%), OB w/o BE = 23 (73.9%), NW = 25 (56%)	BE in children. Sleep in children with obesity	DSM IV BED criteria 1-week Actigraphy, MSQ, Standard Technion Clinical Sleep Questionnaire, Sleep diary	SE% was lower in OB with BE compared to OB w/o BE, and NC groups. WAKE was higher in OB with BE group compared to OB without BE and NC group.	No analysis of association between BE behavior severity and actigraphy or diurnal preference or sleep parameters
Weltzin et al. (81)	CS	NW BN = 24.8 (6.3) NW w/o BN = 22.5 (4.0)	*N* = 65(100%) NW BN = 54, NW w/o BN = 11	Food intake patterns in BN. 24 h hormonal pattern during BE days.	DSM III-TR Cortisol, HGH, PL	Nocturnal prolactin blunted in BN. No significant effect of binging/purging on other hormones.	BE behavior timing and potential effect of compensatory behavior was not analyzed.
Vogel et al. (50)	CS	OB = 43.8(11.2) ADHD = 34.9 (10.6) Controls = 23.6 (3.1)	OB = 114 (86%) ADHD = 202 (47%) Controls = 154 (65%)	Circadian-related variables in ADHD and their relationship with obesity	Dutch version of the ADHD rating scale Morning/Evening type Questionnaire MCTQ	Extreme evening chronotype higher in ADHD vs. OB group. Unstable eating pattern (skipping breakfast + evening/night BE), skipping breakfast and BE behavior at night higher in ADHD vs. OB. Rate of BE behavior similar in ADHD vs. OB. Unstable eating pattern (BE at mediated BMI in ADHD.	No comparisons between ADHD and Control group. No specific analysis for BE behavior association with other clinical variables

ADHD, attention deficit and hyperactivity disorder; AN, anorexia nervosa; BDI, Beck depression inventory; BD, bipolar disorder; BE, binge eating; BED, binge eating disorder; BN, bulimia nervosa; BLT, bright light therapy; CD, circadian; CS, cross-sectional; EAT, eating attitudes test; EDDS, eating disorder diagnostic scale; ED, eating disorder; HDRS, Hamilton depression scale; HGH, growth hormone; Long, longest episode of continuous sleep; MEQ, morningness-eveningness questionnaire; MCTQ, Munich Chronotype Questionnaire; NEQ, night eating questionnaire; NW, normal weight; NE, night eating; OB, obesity; PL, prolactin; PSG, polysomnograph; P, prospective; REAPS, rapid eating assessment for patients; SE, sleep efficiency; TST, total sleep time; w/o, without; Zero, minutes of zero activity counts.

##### Hormonal studies

Three studies have investigated the association between diurnal hormonal variations and BE or BE-like behavior (42, 43). In the first study, normal weight participants with and without BN were evaluated during 72 h in a laboratory setting. Cortisol, growth hormone, and prolactin plasma levels were obtained from blood samples taken every 20 min for 24 h on one of the experimental days. Participants with BN were asked to conduct a BE behavior during one of the days and hormone levels were compared to those without BN. Prolactin levels were decreased during the latter part of the night in patients with BN compared to those without BN. No other differences in 24-h hormonal patterns were observed. The time of BE behavior and presence of purging (e.g., self-induced vomit) or other compensatory behaviors (e.g., vigorous exercise, skipping meals) behavior, as well as sleep/wake cycles were not described. The potential effect of these variables on hormone levels was not analyzed.

In the second study, healthy-weight females (*n* = 7) without a history of an ED were evaluated under a strictly controlled laboratory setting. It was observed that the time of eating determined the diurnal variation of leptin secretion and that consumption of BE-like dinners induced increased fasting glucose levels. Having a BE-like dinner increased postprandial insulin secretion, as well as decreased postprandial leptin secretion without a postprandial difference in glucose levels (43). These findings suggest that consuming BE-like meals might induce profound metabolic changes that persist for hours, potentially altering hormone physiological diurnal variation.

In the third study, 32 individuals with obesity and with (*n* = 16) or without (*n* = 16) BED were evaluated for morning vs. evening variation in fullness and hunger ratings according to visual analog scale ratings, as well as hormonal variation differences in response to a standardized liquid meal or an *ad libitum* buffet-like meal in a controlled environment (42). BED participants showed decreased fullness and a trend toward increased hunger ratings during the evening when compared to control participants in response to the liquid meal. Compared to the control group, BED participants showed decreased fullness after the buffet-like meal in the evening than in the morning and reported an increased sense of loss of control and similarity to a BE behavior episode at both time-of-day conditions. Moreover, altered hormonal patterns were identified in the BED group, with decreased initial fasting ghrelin levels in the morning and increased initial fasting levels in the evening prior to the ingestion of the liquid meal. These findings are consistent with a heightened evening susceptibility to overeating in BED.

##### Actigraphy studies in individuals with binge eating behavior

Five studies employed wrist actigraphy to examine locomotor activity in individuals with BE behavior (44–48). Actigraphy records wrist acceleration and enables objective estimates of behavioral activity, sleep, and the sleep/wake cycle. The first of these studies compared 1-week actigraphy data across three adult groups: individuals with obesity and *DSM-IV* BED (*n* = 18), individuals with obesity and without BED (*n* = 13), and normal weight individuals without BED (*n* = 16) (44). The BED and non-BED obesity groups showed altered actigraphic parameters compared with the normal weight control group, including decreased sleep efficiency (percentage of time asleep while in bed), total sleep time, minutes of zero activity counts, longest episode of continuous sleep, as well as increased total minutes of wake during sleep. There were no differences between BED participants and the obesity control group. Unfortunately, the sample was relatively small and *post hoc* analyses were not clearly described. The second study, conducted in children with obesity (*n* = 36), found that participants with BE behavior (*n* = 13) had a lower sleep efficiency percentage and more minutes of wake during sleep compared with participants without BE behavior (*n* = 23) and a group of normal weight children without BE behavior (45).

The third and fourth studies utilized 5 days of actigraphic recordings to evaluate diurnal variations in adult women with obesity and with or without BED (46, 48). In the third study, BED subjects (*n* = 8) showed lower Midline Estimated Statistic of Rhythm (MESOR; a rhythm-adjusted mean) and absolute amplitude compared with controls (*n* = 8), but no difference in acrophase (time at which the peak of a rhythm occurs) was observed. The fourth actigraphy study (46) reported on a sample expansion from the third study (48). It replicated the MESOR and amplitude findings from the prior study. However, inter-daily stability, a measure of diurnal rhythm strength, was higher in BED (*n* = 14) compared to sex, age, and BMI matched control subjects (*n* = 14). Activity counts on L5 (average activity for the least 5 active hours) and M10 (average activity for the most active 10 h) were lower in BED compared to controls. The results of these two studies are difficult to interpret because BED participants were receiving an intensive (8-h per day) combination of cognitive behavioral therapy and a nutritional program during the 5 days of actigraphic recording that individuals on the control group apparently did not receive. Results may thus indicate a combination between the effects of the intervention inducing better sleep parameters in BED compared with controls subjects and a blunted rest/activity rhythm due to biological differences in patients with BED vs. control, as shown by a decreased MESOR and amplitude.

The fifth study utilized a commercial accelerometer (Fitbit) among other circadian-related assessments (including chronotype and sleep diary and quality parameters) in 52 patients with threshold or sub-threshold *DSM-IV* BN (*n* = 22) or BED (*n* = 30) diagnosed according to the Eating Disorder Diagnostic Scale (EDDS) (47). Global eating psychopathology was not related to sleep duration, sleep efficiency, or sleep onset latency. Participants with BN showed greater ‘eveningness’ and decreased self-reported sleep health compared to the control group, but no difference was observed between the BED and the other groups. Among participants with an eating disorder, greater ‘eveningness’ was strongly associated with increased global eating disorder psychopathology. The main limitation of this study was the inclusion of threshold and sub-threshold BN or BED symptoms in the analysis without providing details about diagnostic criteria distribution. Other limitations included participation of patients in a weight loss program and use of a commercial accelerometer instead of research-designed actigraphy devices.

##### Chronotype (diurnal preference) and binge eating behavior

Three studies evaluated chronotype in individuals with BE behavior (12, 49, 50). In the first study, self-reported ‘eveningness’ was associated with increased severity of BE behavior when assessed with the BE scale among 100 participants seeking care at a nutrition clinic (49). The second and third studies evaluated BE behavior and self-reported diurnal preference in other psychiatric conditions, specifically ADHD (50) and bipolar disorder (12). In the first of these studies, 64% of adult ADHD patients (*n* = 202) had BE behavior, which was comparable to 72% in a group of individuals with obesity (72%, *n* = 114) and higher than a 34% in the control group (*n* = 154) (50). ADHD patients showed a higher rate of proxy markers of circadian phase delay, including self-reported measures of delayed sleep phase syndrome (25.8% vs. 6.9%) and extreme evening chronotype compared with obesity patients (15.5% vs. 4.4%). Of note, ADHD subjects skipped breakfast more often compared to the group of subjects with obesity. Unfortunately, the association between BE behavior and diurnal preference was not reported. However, a similar proportion of subjects with ADHD and obesity reported BE behavior in the evening, but a higher percentage of ADHD subjects reported nocturnal BE behavior compared to the group with obesity. In addition, participants were classified as having an “unstable eating pattern” if they skipped breakfast and had BE behavior at night/evening. Participants with ADHD had a higher rate of unstable eating pattern compared to the group with obesity, but the group with obesity did not differ from controls. It was also observed that the unstable eating pattern mediated body mass index (BMI) only in the ADHD group.

The next study evaluated correlates of self-reported chronotype among 783 patients with bipolar disorder. The 205 (27%) patients with evening chronotype had increased rates of BE behavior, BN, and nocturnal BE behavior, as well as higher EDDS global eating psychopathology scores and higher BMI compared with those with non-evening chronotypes (51). Interestingly, participants with evening chronotype also reported skipping breakfast more often, eating less fruit and vegetables, and consuming more fried foods, unhealthy snacks, and sugary drinks.

##### Circadian system genetics and binge eating behavior

We found one study that investigated circadian system genetics in participants with BE behavior (52). Specifically, the association between the 3111C CLOCK (Circadian locomotor output cycles kaput) allele genotype and obesity in participants with overweight/obesity with BED (*n* = 107) or without BED (*n* = 85) and a normal weight control group (*n* = 92) was evaluated. Secondarily, authors hypothesized that genotypes homozygous or heterozygous for the 3111C CLOCK allele would be associated with obesity compared with the homozygous genotype. Although an association between the 3111T/C genotype and a higher BMI was found, there was no association with BED. This study had several limitations, including the study of a single clock gene, the lack of intermediate or proxy measurements of circadian system function (e.g., chronotype or actigraphy), and lack of reporting on features of BE behavior (e.g., timing, frequency, or severity scales).

#### Overlap of binge eating and night eating behavior

Night eating syndrome is currently included as an example of an “Other Specified Feeding or Eating Disorder” in the *DSM-5* and is defined as recurrent episodes of night eating, manifested by eating after awakening from sleep or by excessive food consumption after the evening meal (1). The behavior causes distress, there is recall and awareness of the behavior, and the behavior is not better explained by another ED.

In contrast to the generally accepted definition of BE behavior, there have been various definitions of night eating behavior and diagnostic criteria for NES. Older studies used Stunkard’s (53) criteria for NES (53), with one criterion being evening hyperphagia, described as consuming 25% or more of the total daily caloric after the evening meal (54). Several food intake and neuroendocrine circadian phase and amplitude disturbances have been documented in NES (55). In some cases, night eating episodes may meet criteria for BE behavior episodes if the amount of food consumed is unusually large and is associated with a sense of loss of control. Conversely, an episode of BE behavior during the night can also meet criteria for a night eating episode.

Eighteen studies evaluated the overlap of BE and night eating behavior. We subdivided these studies into those evaluating the clinical similarities between BE and night eating behavior (*n* = 3), those reporting prevalence estimates of the occurrence of BE and night eating behavior (*n* = 11), and those evaluating clinical correlates of the co-occurrence of these conditions (*n* = 5) (Table 3 and Supplementary Table 4).

**TABLE 3:** Studies on the overlap of BE and night eating behavior.

Author/year	Study design	Participants age (*SD*)	Sample size/Female%	Outcomes	Instruments	Results	Limitations and comments
Adami et al. (60)	P	Bariatric surgery candidates 37.6	*N* = 63/76%	Frequency of BED and NES	*DSM IV* criteria NE defined as no appetite for breakfast, consuming > 50% of food after 7 p.m., and having trouble getting to sleep and/or staying asleep.	BED in 42.8% and NE in 7.9%. All NE patients met criteria for BED.	Standardized tools for BED and NES diagnosis were not used
Allison et al. (58)	CS	BED with OB NES with OB OB w/o BED or NES (Ctrl group) Age per group not provided	BED = 177/79% NES = 68/69% Ctrl = 45/66%	Eating patterns, disordered eating, clinical features, and measures of psychological distress	SCID *DSM IV* for BED EDE NES criteria ≥ 25% caloric intake after evening meal and/or awakenings to eat ≥ 3/week. TFEQ BDI	Higher BDI scores in BED and NES groups compared to Ctrl group. BE behavior frequency BED > NES > Ctrl Objective overeating episodes BED > NES = Ctrl. Breakfast and lunches in NES > BED and Ctrl. Afternoon snacks in NES < BED. Evening meals in BED > Ctrl but no difference with NES. Evening snacks NES > Ctrl. Nocturnal snacks in NES > BED and Ctrl groups. BED and NES groups reported higher dietary restraint and eating concerns than Ctrl. Shape and weight concerns in BED > NES and controls, and NES > controls. Disinhibition and hunger in BED > NES and control groups, and NES > control group.	Study did not include a group of patients with comorbid BED and NES. Depressive symptoms were included as covariate in the analysis of outcome measures.
Allison et al. (62)	CS	Bariatric surgery candidates 44.4 (10.7)	*N* = 215/82%	NES and BED in bariatric pre-surgery patients	*DSM IV*- criteria NEQ	BED in 15% and NES in 3.9% of the total sample. NES in 15% of BED patients.	Homogenous sample. Evening hyperphagia or Nocturnal Eating criteria were not reported separately in BED patients.
Colles et al. (4)	CS	Community sample 41.3 (13.5) OB sample 55.1 (12.4) Bariatric surgery candidate 44.8(11.2)	Total sample = 431 Community *n* = 158/78.5% OB *n* = 93/91.4% Bariatric surgery candidate *n* = 180/78.3%	Clinical significance on NES and nocturnal snacking	Combination *DSM IV* and *DSM 5* criteria for BED Self-report NES survey	BED in 12%, NES in 11%. BED and NES in 4%. NES in 37% of BED. BED in 40% of NES. NES is associated with high BMI and BED. BED seven times more likely to have NES. Significant differences in BED and NES prevalence among sub-samples.	Self-report weight; varying recruitment methods Heterogeneous population
de Zwaan et al. (23)	CS	Community sample reporting Night-time eating 49.2 (?)	N106/64.2%	NE in a community sample	Phone interview. SCID *DSM IV* criteria. NEQ, Night eating syndrome history and inventory, sleep disorder questionnaire, Eating Disorder Questionnaire.	NES criteria 29.2% Evening hyperphagia ≥ 25% or ≥50% calorie intake after evening meal in 76.4 and 45.3% of the sample. No control over NE food ingestion in 44.5% of NES participants. 14.2% had BED or BN	All participants had self-reported night eating. Clinical features of NE episodes among BED or BN with NES participants was not described.
Greeno et al. (57)	P	Women with OB seeking treatment 39.47 for BED 39.07 for Ctrls	DSM III BED *N* = 39/100% Ctrls *N* = 40/100%	Behavioral and psychological correlates of NE	Food intake diary for 5 to 10 days. BED diagnosed with BES score > 17. NE defined as “getting up out of bed during the night to eat.”	NE in 7.5% of participants. All subjects with NE had BED. BED with NE was 15% 5/7 NE episodes described with low perceived control and 4/7 were described as likely BE behavior. NE episode average 639 calories and 41% fat content.	Homogenous sample; women only. Small sample size
Grilo and Masheb (61)	CS	DSM IV BED seeking treatment 44.4 (9.3)	*N* = 207/78.3%	Comparison of BED vs. BED + Night-time eating	SCID *DSM IV* criteria Night-time eating based on EDE (eating after going to sleep ≥ 4 days/past 28 days), EDE, TFEQ, BDI, RSE	28% Night-time eating in BED. More men than women had NE in BED. Women with BED and NE had greater eating, weight and shape concerns than men with BED and NE.	Homogenous sample
Grilo et al. (24)	CS	BED with OB Hispanic/Latino seeking treatment; 46.32 (9.68)	*N* = 79, 81%	NE in obese Hispanic population	SCID DSM IV criteria Night-time eating based on EDE (eating after going to sleep at least once in the past 28 days) EDE, BDI	NE in 53% of BED participants and 23% of participants without BED. BED present in 70% of those without NE and 18% of those without NE. Frequency of BE behavior and NE episodes were correlated NE associated with higher levels of psychopathology	Homogeneous sample. Comparison of clinical characteristics between BED with and without NE groups were not explored.
Harb et al. (49)	CS	Outpatient nutrition clinic 39.5 (11.7)	*N* = 100, 77%	Correlation between chronotype and eating behavior	NEQ, MEQ, BES BE behavior classified as BES ≥ 18	BE behavior in 18% and NE behavior in 18%. BES and NEQ scores were inversely correlated with MEQ; BES and NEQ were also associated	Self-report instruments. Co-occurrence of BE and NE behaviors not reported. BED criteria not used for analysis.
Latzer et al. (5)	P	Women seeking treatment with BE behavior (BN or BED) with NES or without NES	*N* = 59/100% BE behavior with NES = 25 BE behavior without NES = 34	Dietary pattern differences between those with NES and without NES	*DSM IV* criteria 24 hour food diary (7 days) BDI	Participants with NES had higher BDI scores, more BE behavior days and episodes per week, calories ingested per day, and higher evening consumption of calories, than those without NES	BE group included BN and BED patients. Characteristics of NE episodes and timing of BE behavior was not reported.
Meule et al. (69)	CS	German college students 23.55 (3.89)	*N* = 729/77%	Correlation between NE, BMI, emotional eating and binge eating	Online NEQ, MES, EDE	NE, BE, emotional eating and BMI are positively correlated. NE severity related to more frequent BE	Self-report; normal weight sample. Diagnostic for BE, BED or NES prevalence not reported
Napolitano et al. (67)	CS	Weight loss program participants 48.1	*N* = 83, 52.5%	Psychological and behavioral characteristics associated with both NES and BED	*DSM IV* criteria QEWP-R, TFEQ, RSE, ESES, CES-D, NESI, IDED-IV	No ED = 27%; BED = 15%; NES 27%; BED + NES = 15% NES scored lower on disinhibition than BED. BED + NES scored higher on state and trait anxiety and disinhibition, than NES alone	Homogenous sample of participants planning a 4 week stay at weight loss program. Small sample size.
Rand et al. (59)	CS	General sample 52.8 (19.8) Post bariatric restriction surgery patients 44.6 (10.4)	Non-clinical sample (*n* = 2115)/57.5% Post bariatric surgery (*n* = 111)/93.1%	Prevalence of NES	Self-report for NE symptoms. NES: presence in the past 2 months of all the followings: morning anorexia, delay of eating after awakening for several hours, excessive evening eating, evening tension and/or feeling upset, and insomnia.	1.5% NES in general sample; 27%NES in patient sample; 26% NES patients reported BE behavior	Self-report.
Root et al. (65)	CS	Swedish twin study registry	Twins *N* = 11,604 BE = 427/92% NE = 419/54%	Heritability of BE and NE behavior	Online survey with DSM-IV criteria for BE and two independent questions for NE.	Heritability estimates for BE were 0.74 and for NE were 0.35. Genetic correlation between BE and NE behavior was 0.66.	Self-report online questionnaires. Low male BE prevalence.
Runfola et al. (66)	CS	University Students 20.9 (1.7)	Total = 1,636/59.5%	NES prevalence and characteristics in University Students	BE based on EDE DSM-5 criteria and ≥4/past month NES based on NEQ ≥ 25 (broad) or ≥ 30	NES in 4.2% 32.8% on the NES group had BE Participants with NES had 4x BE behavior episodes compared to those without NES (4.4 vs. 1.4 episodes/past month)	Self-reported evaluation 60% were competitive athletes
Sassaroli et al. (68)	CS	Patients with obesity previously admitted to ED unit 48.5 (12.9)	Total *N* = 202/80% BED = 54/? NES = 13/? BED + NES = 16/?	Nocturnal anxiety severity in BED, NES or both	DSM IV TR criteria for BED NES Dx established by eating ≥ 25% daily food intake after dinner, to be affected by morning anorexia and nocturnal awakenings followed by nocturnal ingestions at least 2/week for 3 months. NEQ, SAS, SDQ	Severe anxiety in BED + NES; Correlation between SAS and nocturnal ingestions in BED; Evening hyperphagia correlated to nocturnal mental anxiety in NES and with daytime mental anxiety in BED	Self-reported evaluation with SAS and SDQ
Schenck et al. (56)	CS	Sleep-related eating Disordered Adults 38.8 (9.8)	*N* = 38, 65.8%	Eating behavior and clinical characteristics of sleep-related eating	Clinical interview SCID DSM-III-R Daytime eating disorders questionnaire	68% reported high caloric nocturnal binging 84% reported nightly sleep-related binge eating (without hunger or purging).	Small sample size; self-report. Nightly sleep-related binge eating data only reported in abstract and not clearly described in results section.
Striegel-Moore et al. (63)	CS	Community sample of insured women age range 18–35	*N* = 259 BED *N* = 89 Ctrls	Clinical correlation of NE in BED vs. no BED	BED defined as episodes of overeating with LOC at least once/week in the past 3 months. NE based on EDE (at least 1 NE episode in the past 28 days). Survey questions, BDI, RSE, WSAS	NE in 12.5% of the total sample. % of NE participants with at least 1 BE behavior and with recurrent BE behavior (1/week for the past 3 months) was higher compared to no NE group (39% vs. 20.8% and 26.8% vs. 11.1%, respectively).	Homogenous sample of white female
Tholin et al. (64)	CS	Population based sample of Swedish twins 37.4 (7.5)	*N* = 741/55%	NE in twin samples	Survey questions	NE is associated with BE, sleep disturbance and obesity	LOC not assessed for NE

BDI, Beck Depression Inventory; BE, binge eating; BED, binge-eating disorder; BES, binge eating scale; BMI, body mass index; bn, bulimia nervosa; CES-D, center for epidemiological studies depression scale; ctrl, control; CS, cross-sectional; DSM, diagnostic and statistical manual of mental disorders; ED, eating disorder; EDE, eating disorder questionnaire; F, female; IDED-IV, 4th ed. of the interview for diagnosing eating disorders; MEQ, morningness-eveningness questionnaire; LOC, loss of control; MES, mood eating scale; NE, night eating; NEQ, night eating questionnaire; NES, night eating syndrome; NESI, night eating syndrome interview; OB, obesity; P, prospective; QEWP-R, questionnaire on eating and weight patterns revised; RSE, rosenberg self-esteem scale; SAS, self rating anxiety scale; SCID, structured clinical interview for DSM; SD, standard deviation SDQ, self rating anxiety scale; TFEQ, three factor eating questionnaire; WSAS, work and social adjustment scale.

##### Clinical overlap between binge eating and night eating behavior

The first of three studies exploring clinical similarities between BE and night eating behavior evaluated 38 patients with sleep related eating conditions (56). Although no patient was diagnosed with daytime BE behavior, 26 (68.4%) reported “high caloric nocturnal binging” and 27 (71%) reported night sleep-related BE (without hunger or purging). The authors noted that sleep-related eating episodes were characterized by the immediate and “compulsive” urge to eat as well as the tendency to eat high calorie foods (e.g., milkshakes, pies, brownies, and candy). These foods were not typically consumed during the day. In the second study, the presence and characteristics of night eating episodes were analyzed in 40 patients with BED and obesity during a 1-week follow-up period (57). Six (15%) patients had at least one night eating episode with an average of 639 calories with 41% fat content. Four out of the seven documented night eating episodes were described as “definitely or sort of a BE episode,” and five out of seven were described as being associated with lack of control during food ingestion. Perceived low control over eating during night eating episodes was highly correlated with describing the night eating episode as being a BE behavior episode.

A third study evaluated the clinical features of participants with BED (*n* = 177), NES (*n* = 68), and a control group of individuals with overweight or obesity without BED or NES (*n* = 45) (58). The BED and NES groups had elevated depressive symptom scores compared to the control group and therefore depression scores were used as a covariate, along with BMI, in analysis comparing eating-related behaviors. As expected, BE behavior frequency was higher in the BED group compared to the other two groups and BE behavior frequency was also higher in the NES group compared to the control group; unfortunately, timing of BE behavior was not reported. The BED group reported more objective overeating episodes and more frequent eating episodes during the day than both the NES and control groups. The three groups differed significantly in several food intake patterns. NES participants reported fewer breakfast and lunches compared to the BED and control groups, whereas the BED group reported more afternoon snacks compared to the NES group. The BED and NES groups had more evening meals and evening snacks compared to the control group but did not differ from each other. The NES group reported more nocturnal snacks compared to BED and control groups. BED and NES groups reported higher dietary restraint and eating concerns than the control group. Disinhibition and hunger were higher in BED than in the NES and control groups and were higher in NES compared to the control group.

##### Prevalence estimates of co-occurrence of binge eating and night eating behavior

Eleven studies reported estimates of the co-occurrence of BE and night eating behavior among clinical (*n* = 7) and non-clinical (*n* = 4) samples. The first clinical study evaluated 111 bariatric surgery patients and found NES present in 30 (27%) of the sample. Eight (27%) of those patients with NES also had BE behavior (59). The second study evaluated patients eligible for bariatric surgery for both *DSM-IV* BED and NES. Of 63 patients, 27 (42.8%) met BED criteria and 5 (7.9%) met criteria for NES (60). Importantly, all NES patients met criteria for BED, while none of those without a history of BE behavior had a history of night eating behavior. In the third study, which evaluated 106 participants with self-reported night eating behavior, 31 (29.2%) met full criteria for NES [Stunkaard’s (53) criteria], and 13 (12%) and 21 (20%), respectively, met criteria for “current” or “lifetime” BED or BN by *DSM-IV* criteria (23).

The fourth study evaluated 207 treatment-seeking patients with *DSM-IV* BED and found that 58 (28%) had at least one episode of “night-time eating,” defined as eating after having gone to sleep, in the past 28 days (61). Night eating episodes occurring as frequently as half of the past 28 days was reported by 19 (9.2%) of BED participants. BED participants with NE showed a higher current and lifetime BMI compared to those without night eating. Both groups were similar regarding BED age of onset and other eating behavior and mood related clinical characteristics. Rate of night eating behavior was higher in men (42%) than women (24%), but women with night eating had higher eating, body shape, and weight concerns scores on the Eating Disorders Examination subscales than their male counterparts.

A fifth study with 215 bariatric surgery eligible participants with obesity, reported 15% had BED (*DSM-IV* criteria) but only 3.9% of all participants met criteria for NES based on the night eating questionnaire (NEQ) that included both evening hyperphagia and nocturnal food ingestion (62). However, 23.7% showed evening hyperphagia only and 7.2% reported nocturnal food ingestion only. The authors also conducted a clinical interview evaluating eating behavior in the past 2 days and found that NES prevalence was reduced to 1.9%. Among 27 patients that met criteria for NES or BED according to a clinical interview, 18.5% (*n* = 5) met criteria for both.

The sixth study evaluated 431 individuals, including 93 from a weight loss program, 180 eligible for bariatric surgery, and 158 from the community (4). BED criteria (*DSM IV* or *DSM 5* criteria equivalent) were met by 12% of the total sample. NES criteria (based on a past 3-month self-report survey) were met by 11% of participants. In total, 4% met criteria for BED and NES. Overall, 37% of participants with BED met NES criteria. On the other hand, 40% of NES participants also met criteria for BED. Of note, participants with BED were seven times more likely to meet criteria for NES. The seventh study investigated 79 treatment-seeking Hispanic patients with obesity with BED (*n* = 40) or without BED (*n* = 39) and reported that 38% of the total sample met criteria for regular night eating episodes (≥4 days/month) (24). Night eating was present in 53% of participants with BED, and only in 23% of those without BED. Conversely, BED criteria were met by 70% of participants with night eating versus 18% of those without night eating. Frequency of BE behavior and night eating episodes was directly correlated.

In the first non-clinical study, 41 (14.3%) of 285 women from the community reported night eating episodes (63). Having at least one BE episode or having recurrent BE behavior episodes in the past 28 days was reported by 39 and 26.8% of participants with night eating, which was higher than the 20.5 and 11.1% of those without night eating, respectively. The second study evaluated a population-based sample of 21,741 twins (zygosity not reported) and found the prevalence of night eating was 4.6% among men and 3.4% among women (64). Men and women with night eating had 3.4- and 3.6-times higher risk of BE behavior, respectively, compared to those without night eating. In addition, night eating was 2.5 and 2.8 times higher in men and women with obesity, respectively, compared with normal weight men and women. No twin-pair data was reported.

The third study evaluated heritability of BE and night eating behavior in 11,604 Swedish twins (mono and dizygotic pairs) (65). Heritability estimates were 0.74 for BE behavior and 0.35 for night eating. The study also reported a genetic correlation of 0.66 between BE and night eating behaviors, suggesting significant genetic overlap.

The fourth study evaluated NES and BE behavior among 1,636 university students (66). NES criteria were met by 67 (4.2%) participants, of which 22 (32%) also reported BE behavior. Compared to those without NES, individuals with NES had almost four times more BE behavior episodes per month.

##### Clinical features of co-occurrence of binge eating and night eating behavior

Five studies evaluated the associated clinical features in patients with both BE and night eating behavior. The first study evaluated 83 patients seeking weight loss treatment. Thirteen (15%) had *DSM-IV* BED, 23 (27%) had NES (based on two questions), 13 (15%) had BED and NES, and 27% did not meet criteria for an ED (*n* = 34). Participants with BED and NES showed greater behavioral disinhibition and trait and state anxiety as compared to those with NES alone (67). The second study found that self-reported anxiety was higher in patients with BED and NES as compared to those with only BED and those with only NES (68).

The third study examined chronotype in relation to BE and night eating behavior among 100 patients attending a nutrition clinic (49). Significant correlations were found between BE and night eating severity scores and self-reported diurnal preference assessments, suggesting that eveningness was associated with higher severity of both BE and night eating behavior. Moreover, BE and night eating severity scores were significantly correlated. The fourth study identified a positive correlation between BE behavior frequency and severity of night eating behavior among 729 college students (69). The fifth study investigated dietary pattern differences between patients with *DSM-IV* BE behavior with NES (*n* = 25) or without NES (*n* = 34) during a 1-week prospective evaluation (5). Participants with NES had higher scores on depressive symptoms, more BE behavior days and episodes per week, ingested more calories per day, and had a higher consumption of calories after the evening meal compared to those without NES.

### Interventional studies

**FIGURE 2:**
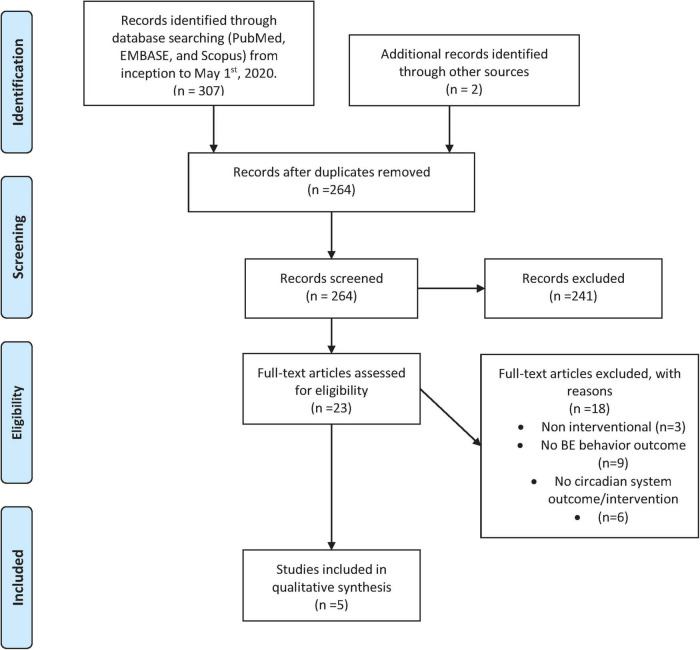
PRISMA flowchart for interventional studies.

Five relevant interventional studies were identified (see Figure 2, Table 4 and Supplementary Figures 1, 2). All studies evaluated bright light therapy (BLT) in patients with BN and reported outcomes on BE behavior. No studies examining melatonin, melatonergic agents, or scheduled food intake were identified.

**TABLE 4:** Interventional studies.

Author, year	Study design	Participants, Age	Sample size/Female%	Intervention	Outcomes	Instruments	Results	Limitations and comments
Blouin et al. (74)	1- week Double-Blinded RCT	BN, 27.9 (8.0)	BN = 18/100% BLT = 9, Dim light = 9	Laboratory sessions: Fluorescent “BLT” (2,500 lux) or “Dim light” (500 lux) at 3 ft. from source at 17:00 h. 2 h/session, 6 days	BE behavior episodes Compensatory behavior Depressive symptoms	NIMH-DIS-R BDI SIGH-SAD	No group difference in BE episodes, compensatory behavior, perceived control of food intake, or perceived control of food intake. BLT group decreased BDI scores compared to Dim light group.	Short study duration. BLT administered in the evening, at low intensity, and large distance from source (3 ft).
Braun et al. (70)	3- week Double-Blinded non-randomized controlled trial	BN, BLT = 30.0 (7.3) Dim light = 30.5 (8.6)	BN = 34/100% BLT = 16, Dim light = 18	BLT (10,000 lux) or red dim light (50 lux) for 30 min, administered between 6-9 am, for 3-weeks	Assess change in: BE episodes Compensatory behavior Depressive symptoms	SCID-*DSM IV* Eating behavior Diaries DEBQ HAM-D	Greater BE/week decrease in BLT vs. Dim light group. No group difference at follow-up. No group differences in compensatory behavior, meal or snack frequency, or urge to binge carbohydrates, or HAM-D scores.	Short study duration. MDD in 22-25% of participants Distance from light source during sessions not reported
De Young et al. (73)	2- week open-label	BN = 23.58 (4.52)	BN = 9/100%	BLT (10,000 lux) for 30 min, between 7-8 AM	Assess relationship between change in BE episodes, Compensatory behavior, and negative affect	SCID- *DSM IV*-TR EDE CHEDS CESD-R	CHEDS BE scale decreased during intervention. 27% decrease in BE days. Negative affect does not account for BE change. No significant decrease in compensatory behavior.	Short study duration BE days/week not reported BE/week change not reported Distance from light source during sessions not reported
Lam et al. (71)	2-weeks double blinded, randomized controlled crossover design	BN, 31.6 (6.5)	BN = 17/100%	Early morning (7:00 to 8:00 am) BLT (10,000 lux) or Dim red light (500 lux) for 30 min	BE episodes Compensatory behavior Depressive symptoms	*DSM-III* criteria Eating disorders diaries Eating Attitudes Test HAM-D	BLT decreased BE and compensatory behavior compared to dim light intervention. Difference in Eating Attitudes Test approached significance. BLT decreased depressive symptoms according to HAM-D, BDI.	Short study duration, small sample. Distance from light source not described. Fixed sleep schedules (10:00 pm to 7:00 am) and early morning session timing could induce phase advance through forced wake up time. Quick “relapse” of BE after BLT crossover to dim light.
Lam et al. (72)	4-week open-label	BN + MDD (SAD) = 30.2 (5.5)	BN + MDD (SAD) = 22/100%	BLT (10,000 lux) for 30-60 min, between 7–9 am, for 4 weeks	BE episodes Compensatory behavior Depressive symptoms	Eating behavior diaries HAM-D BDI	Decrease in BE and compensatory behaviors Decrease in depressive symptoms	45% taking antidepressant medication for at least 4 weeks Distance from light source during sessions not reported

BDI, Beck depression inventory; BE, binge eating; BLT, bright light therapy; BN, bulimia nervosa; CESD-R, Center for Epidemiological Studies Depression Scale – Revised; CHEDS, Change in Eating Disorder Symptoms Scale; DEBQ, Dutch Eating Behavior Questionnaire; DSM, Diagnostic and Statistical Manual of Mental Disorders; HAM-D, Hamilton Depression Rating Scale; MDD, Major Depressive Disorder; NIMH-DIS-R, National Institute of Mental Health Diagnostic Interview Schedule-Revised; RCT, randomized controlled trial; SAD, seasonal affective disorder; SCID, structured clinical diagnostic interview for the DSM; SIGH-SAD, Structured Interview Guide for the Hamilton Depression Rating Scale-Seasonal Affective Disorder Version.

In four studies, including one non-RCT with a parallel group design (70), one RCT with a crossover design (71), and two open-label trials (72, 73), BLT was administered in the morning (between 6 and 9 AM) at 10,000 lux for 30–60 min. In the parallel-group clinical trial, a statistically significant decrease in BE behavior frequency was reported after three weeks in the active BLT group compared with the sham BLT group (70). However, there was no significant group difference in compensatory behavior. In the crossover RCT, the active BLT group showed a statistically significant decrease in the frequencies of both BE and compensatory behaviors after two weeks as compared to the sham group (71). In a 4-week open label study in participants with BN and a comorbid seasonal affective disorder, BLT decreased BE and compensatory behavior frequency (72). More recently, a 2-week open label study reported a decrease in BE-behavior frequency with BLT but with no change in compensatory behaviors (73). Of note, none of these studies described the distance between the subject and the light source, which is helpful to ensure the intensity of light to which each subject is actually exposed remains constant throughout light sessions.

In the only study that administered BLT in the evening, 18 patients were randomized to receive 2 weeks of bright (2,500 lux, *n* = 9) or moderate (500 lux, *n* = 9) light, each given at 17:00 h for 2 h and at three feet from the light source (74). There were no significant differences between the active or sham groups regarding frequencies of BE or compensatory behaviors, or in perceived control of food intake.

Taken together, these findings suggest that morning BLT (e.g., at 10,000 lux) might reduce BE behavior. However, these data must be considered preliminary due to small sample sizes and heterogeneity in study design and BLT characteristics.

## Discussion

Binge eating behavior is a well-defined trans-diagnostic psychopathological phenomenon with clinical features that suggest a potential circadian system involvement. Although little systematic research has directly assessed the chronobiological aspects of BE behavior, we identified four broad categories of studies: (1) studies of patterns of food intake and/or timing of BE behavior (*N* = 16); (2) studies of circadian-related measures among individuals with BE behavior (*N* = 11); (3) studies of the co-occurrence of BE behavior and night eating (*N* = 18); and (4) studies evaluating BLT in the treatment of BN patients and reporting outcomes on BE behavior (*n* = 5). Studies of food intake timing typically found that BE behavior, as well as higher caloric intake in individuals with BE behavior, occurred later during the day. Studies on circadian-related measures found that BE behavior is associated with diurnal hormone and locomotor activity disturbances, as well as a late diurnal preference or more “eveningness.” Studies of BE and night eating behavior found substantial overlap between these constructs. Interventional studies, though limited, suggest that morning BLT might reduce BE behavior. Below, we summarize and discuss the primary findings of the studies and identify research gaps in each category.

### Patterns of food intake and timing of binge eating behavior

Evidence from studies in this category suggest a delayed food intake pattern and BE behavior occurrence throughout the day in individuals with BE behavior. A lower caloric consumption in the morning and a higher caloric consumption later in the day, may be a common trait among individuals with BE behavior that is stable during BE and non-BE behavior days (15, 28). These observations are consistent with the reports indicating that BE behavior and the urge to binge eat occur predominantly in the later part of the day (16, 36–39). In addition, irregularity and quantity of evening meal or snack consumption may be associated with increased frequency of BE behavior (29–34), and this association may be influenced by several factors including photoperiod (40) and cultural differences (35). However, it is also important to consider that the observed food intake patterns and timing of BE behavior could result from an interaction between chronobiological and non-chronobiological factors, or merely non-chronobiological factors. For example, the effect of environmental factors such as societal and work-related schedule restrictions, the associated homeostatic pressure on hunger and appetite regulatory mechanisms and how these may affect the timing and patterns of food intake is not known. Moreover, inverted sleep/wake cycles (e.g., shift work) or nighttime exposure to light may be associated with disturbances in circadian system function (75–77) that impact eating behavior in unknown ways and warrant further study (76, 78–80).

### Assessment of circadian system function among individuals with binge eating behavior

This category was characterized by a limited number of studies with objective circadian-related assessments and no studies utilizing best-established techniques to assess circadian system function, and an involvement of the circadian system in BE behavior is not yet unequivocally confirmed. However, metabolic changes after binge-like food ingestion or BE behavior (42, 43, 81), actigraphic changes in subjects with BE behavior (44–47), as well as the potential association between evening chronotype and BE behavior (12, 49) support that aspects of the circadian system may be potentially compromised in affected individuals. Of note, studies reviewed have important limitations. For example, interpretation of earlier studies is challenging because subjects with BED were not typically included or BE behavior was often not examined independently from compensatory behaviors. Additionally, most studies are small with a preponderance of female participants and rely on retrospective self-report measures subject to response bias (e.g., underreporting of BE behavior and disordered eating symptoms). Early studies also had limited means of measuring circadian-related parameters. More recently and despite its limitations (e.g., inaccurate sleep onset time assessment), wrist actigraphy has become a useful circadian-related parameter. However, more studies with longer actigraphic recordings that prospectively document food intake pattern and BE behavior timing are needed to characterize locomotor activity in BE behavior. Future studies will be needed to address these limitations as well as to take advantage of the improved availability of objective measures of circadian system function (e.g., dim light melatonin onset) to prospectively study the chronobiology of BE behavior.

Of note, genetic research on the topic is extremely limited (52, 82), and we did not find genome-wide association studies on BE behavior and the circadian system. Whether the potential association between BE behavior and circadian system function is mediated by genetic factors is currently unknown. In addition, studies on the circadian system function and BE behavior in youth are lacking. This is a relevant knowledge gap because BE behavior may occur early in life when the circadian system is still maturing. Understanding if BE behavior is associated with anomalies in the developmental trajectory of the circadian system may provide valuable insight into the normative-pathological spectrum of eating behavior and should be addressed.

### Overlap of binge eating and night eating behavior

As observed in individuals with BE behavior, night eating and NES are characterized by a delayed pattern of food intake, suggestive of circadian system involvement which adds to their substantial clinical overlap (4, 23, 56, 57, 59, 60, 62–64, 67) and potential shared heritability (65). Moreover, individuals with both BED and NES appear to have higher BMI or levels of psychopathology compared to individuals with BED without night eating (4, 24, 61, 64, 68). However, there is a significant knowledge gap regarding the co-occurrence of BE behavior in night eating episodes and how to differentiate the two conditions as the timing of BE behavior, amount of food intake, and loss of control in night eating is not systematically documented in most studies. In addition, most BE behavior episodes occur in the later part of the day but BE is not usually investigated in night eating or NES, or considered as a possible comorbidity.

It remains unclear if BE behavior during the day differs from BE behavior during the night, how often night eating episodes meet strict criteria for BE behavior, and whether the circadian system might be implicated and how is not known. Presently, NES criteria do not consider the absence or presence of BE behavior during a night eating episode. Moreover, NES DSM-5 criteria under “Other Specified Feeding or Eating Disorder” limits its diagnosis if “the disordered pattern of eating is not better explained” by BED or another mental disorder…” (1). This perpetuates a notion of BE behavior and night eating as mutually exclusive phenomena, which has no clinical or scientific basis. Moreover, this may interfere with the identification and study of the association between BE behavior and night eating in the clinical and research settings that hampers the understanding of the potential role of the circadian system in both.

### Interventional studies

This category included five studies evaluating BLT in patients with BN. BLT administered in the morning appeared to decrease BE behavior (70–73), an effect that was not observed in the one study administering BLT in the evening (74). BLT directly targets the circadian system, and these results suggest that a phase advance (circadian shift to occur earlier) or entrainment of the circadian system may influence BE behavior. These studies had a number of methodological limitations that curtail their clinical applicability, and their findings must be considered preliminary. In addition, it is not possible to discard a publication bias on this field. Nonetheless, BLT and other chronobiological interventions, such as nightly administration of exogenous melatonin or melatonin analogs and scheduled sleep/wake cycles, meals, or exercise, may require further consideration in the management of individuals with BE behavior (83, 84).

## Potential future areas of research

Despite recent advances in the understanding on how the circadian system may be involved in appetite/hunger and metabolic regulation, knowledge of its potential role in EDs lags behind (8, 10, 85–87). Virtually none of the assessments that have been performed to date can distinguish involvement of the circadian system vs. influences of the sleep/wake, rest/activity, dark/light, fasting/eating cycle, mood/impulse control rhythms, and/or social/work/school schedules in causing daily rhythms related to BE behavior. It is thus relevant to highlight additional knowledge gaps and areas of opportunity in this field.

First, the concept of BE behavior as a discrete phenomenon has only recently gained the attention of clinicians, researchers, and the pharmaceutical industry. Exploring stand-alone BE behavior time-of-day occurrence and associated food intake patterns might serve to provide valuable insight into its relationship with the circadian system in clinical studies and preclinical research models (7, 88). Second, the current approach to studying the co-occurrence of BE behavior and night eating requires further attention. A step forward could be the recognition that a night eating episode may or may not meet criteria for BE behavior, and that this form of “night BE behavior” episodes require proper assessment, documentation, and analysis. Greater understanding of these issues might challenge the notion that NES is limited by the presence of other EDs as currently conceptualized in the DSM-5 (1). Third, the use of diaries to assess BE behavior and food intake patterns, combined with the use of best-established techniques (e.g., CR or FD) and objective tools (e.g., dim light melatonin onset) to assess circadian system function in BE behavior could contribute to address the indirectness (use of proxy measures) and enhance certainty of published evidence to close the knowledge gap on the association between circadian system function and BE behavior. Fourth, as supported by the available interventional studies (70–73), chronobiological interventions (e.g. morning BLT, evening melatonin) to induce circadian phase advances to assist with realignment in subjects with BE behavior may provide a mechanistic tool to further understand the potential influence of the circadian system in BE behavior, and to evaluate whether the circadian system represents a plausible therapeutic target.

Finally, understanding the mechanisms by which certain medications that affect BE behavior may also act on the circadian system may provide important clues. For example, antipsychotics may have a deleterious effect on BE behavior but their effect on the circadian system is poorly understood (89, 90). Likewise, there are medications used to reduce BE behavior for which an effect on the circadian system is plausible but currently unexplored, for example, lisdexamfetamine and topiramate (91). However, BE behavior clinical trials seldom include the assessment of outcome measures that inform on circadian system function. Perhaps it is time for that to change.

## Summary

**FIGURE 3:**
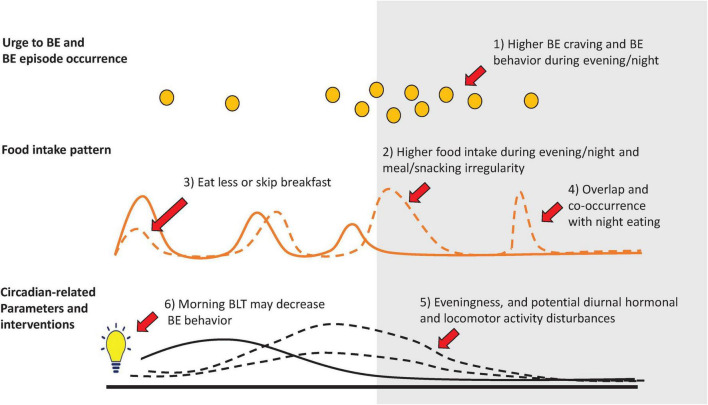
Circadian-related features in binge eating (BE) behavior. Illustrates six key circadian-related features that support a potential circadian system involvement in BE behavior: (1) Although both, the urge to binge eat and BE behavior (orange circles) may occur at any time of day (e.g., during a one-month period), they occur predominantly during the late afternoon/evening and night periods. Compared to individuals without BE behavior (solid lines), food intake in individuals with BE behavior (dashed lines) is characterized by; (2) higher calorie intake during the evening/night, and BE behavior frequency/severity is associated with late meal/snack irregularity, (3) less food intake in the morning or skipping breakfast more often, and (4) show clinical overlap and co-occurrence with night eating behavior. Circadian-related parameters associated with BE behavior include: (5) late diurnal preference (eveningness), as well as potentially disturbed diurnal hormonal and locomotor activity parameters, and (6) a potential therapeutic effect of morning bright light therapy (BLT) in BE behavior. Background colors illustrates day to the right (white) or evening/night periods to the left (gray).

We identified six key findings that support the need to further explore the circadian system in BE behavior and its therapeutic potential. These are: (1) BE craving and behavior tend to occur in the later part of the day; (2) infrequent food intake and/or snacking during the evening/night are associated with increased BE behavior; (3) individuals with BE behavior tend to eat less early in the day or to skip breakfast; (4) BE behavior frequently co-occurs with night eating with remarkable clinical overlap; (5) eveningness, as well as diurnal hormonal and locomotor activity abnormalities, may be associated with BE behavior; and (6) interventions that specifically target the circadian system may decrease BE behavior (Figure 3). A continued effort to study the chronobiological aspects of BE behavior will be necessary to advance this re-emerging and relevant topic.

## Author contributions

FR-N, AG, NM, and SM conceived and/or designed the work. FR-N, AG, NM, FL, CG, and SM conducted the primary and/or secondary literature search, data extraction, synthesis, and interpretation. FR-N, AG, NM, FS, HB, RM, JW, CG, and SM played an important role in interpreting the results. All authors contributed to drafting and revising the manuscript, and approved the final version.

## References

[1] American Psychiatric Association. *Diagnostic and Statistical Manual of Mental Disorders.* 5th ed. Virginia, VA: American Psychiatric Association (2013). 10.1176/appi.books.9780890425596

[2] Baek JH Kim K Hong JP Cho MJ Fava M Mischoulon D Binge eating, trauma, and suicide attempt in community adults with major depressive disorder. *PLoS One.* (2018) 13:e0198192. 10.1371/journal.pone.0198192 29927937 PMC6013207

[3] Lydecker JA Grilo CM. Psychiatric comorbidity as predictor and moderator of binge-eating disorder treatment outcomes: an analysis of aggregated randomized controlled trials. *Psychol Med.* (2021) 14:1–9. 10.1017/S0033291721001045 33849682 PMC8514588

[4] Colles SL Dixon JB O’Brien PE. Night eating syndrome and nocturnal snacking: association with obesity, binge eating and psychological distress. *Int J Obes.* (2007) 31:1722–30. 10.1038/sj.ijo.0803664 17579633

[5] Latzer Y Yutal AE Givon M Kabakov O Alon S Zuckerman-Levin N Dietary patterns of patients with binge eating disorders with and without night eating. *Eat Weight Disord.* (2020) 25:321–8. 10.1007/s40519-018-0590-2 30327996

[6] Buijs FN Leon-Mercado L Guzman-Ruiz M Guerrero-Vargas NN Romo-Nava F Buijs RM. The circadian system: a regulatory feedback network of periphery and brain. *Physiology.* (2016) 31:170–81. 10.1152/physiol.00037.2015 27053731

[7] Buijs RM Soto Tinoco EC Hurtado Alvarado G Escobar C. The circadian system: from clocks to physiology. *Handb Clin Neurol.* (2021) 179:233–47. 10.1016/B978-0-12-819975-6.00013-3 34225965

[8] Scheer FA Morris CJ Shea SA. The internal circadian clock increases hunger and appetite in the evening independent of food intake and other behaviors. *Obesity.* (2013) 21:421–3. 10.1002/oby.20351 23456944 PMC3655529

[9] Mason IC Qian J Adler GK Scheer F. Impact of circadian disruption on glucose metabolism: implications for type 2 diabetes. *Diabetologia.* (2020) 63:462–72. 10.1007/s00125-019-05059-6 31915891 PMC7002226

[10] Chellappa SL Qian J Vujovic N Morris CJ Nedeltcheva A Nguyen H Daytime eating prevents internal circadian misalignment and glucose intolerance in night work. *Sci Adv.* (2021) 7:eabg9910. 10.1126/sciadv.abg9910 34860550 PMC8641939

[11] Chellappa SL Vujovic N Williams JS Scheer F. Impact of circadian disruption on cardiovascular function and disease. *Trends Endocrinol Metab.* (2019) 30:767–79. 10.1016/j.tem.2019.07.008 31427142 PMC6779516

[12] Romo-Nava F Blom TJ Guerdjikova A Winham SJ Cuellar-Barboza AB Nunez NA Evening chronotype, disordered eating behavior, and poor dietary habits in bipolar disorder. *Acta Psychiatr Scand.* (2020) 142:58–65. 10.1111/acps.13179 32335894

[13] Kandeger A Egilmez U Sayin AA Selvi Y. The relationship between night eating symptoms and disordered eating attitudes via insomnia and chronotype differences. *Psychiatry Res.* (2018) 268:354–7. 10.1016/j.psychres.2018.08.003 30098543

[14] Gamsizkan Z Onmez A Sahip Karakas T. Chronobiological evaluation and an intervention study on timing of food intake in the treatment of obesity. *Int J Clin Pract.* (2021) 75:e14502. 10.1111/ijcp.14502 34117692

[15] Raymond NC Neumeyer B Warren CS Lee SS Peterson CB. Energy intake patterns in obese women with binge eating disorder. *Obes Res.* (2003) 11:869–79. 10.1038/oby.2003.120 12855757

[16] Schreiber-Gregory DN Lavender JM Engel SG Wonderlich SA Crosby RD Peterson CB Examining duration of binge eating episodes in binge eating disorder. *Int J Eat Disord.* (2013) 46:810–4. 10.1002/eat.22164 23881639 PMC3889648

[17] Beauchamp MT Lundgren JD. A systematic review of bright light therapy for eating disorders. *Prim Care Companion CNS Disord.* (2016) 18. 10.4088/PCC.16r02008 27835724

[18] Scheer F Hilton MF Evoniuk HL Shiels SA Malhotra A Sugarbaker R The endogenous circadian system worsens asthma at night independent of sleep and other daily behavioral or environmental cycles. *Proc Natl Acad Sci U.S.A.* (2021) 118:e2018486118. 10.1073/pnas.2018486118 34493686 PMC8449316

[19] Damiola F Le Minh N Preitner N Kornmann B Fleury-Olela F Schibler U. Restricted feeding uncouples circadian oscillators in peripheral tissues from the central pacemaker in the suprachiasmatic nucleus. *Genes Dev.* (2000) 14:2950–61. 10.1101/gad.183500 11114885 PMC317100

[20] Garaulet M Lopez-Minguez J Dashti HS Vetter C Hernandez-Martinez AM Perez-Ayala M Interplay of dinner timing and MTNR1B type 2 diabetes risk variant on glucose tolerance and insulin secretion: a randomized crossover trial. *Diabetes Care.* (2022) 45:512–9. 10.2337/dc21-1314 35015083 PMC8918262

[21] Moher D Liberati A Tetzlaff J Altman DG Group P. Preferred reporting items for systematic reviews and meta-analyses: the PRISMA statement. *PLoS Med.* (2009) 6:e1000097. 10.1371/journal.pmed.1000097 19621072 PMC2707599

[22] Peters MD Godfrey CM Khalil H McInerney P Parker D Soares CB. Guidance for conducting systematic scoping reviews. *Int J Evid Based Healthc.* (2015) 13:141–6. 10.1097/XEB.0000000000000050 26134548

[23] de Zwaan M Roerig DB Crosby RD Karaz S Mitchell JE. Nighttime eating: a descriptive study. *Int J Eat Disord.* (2006) 39:224–32. 10.1002/eat.20246 16511835

[24] Grilo CM Milsom VA Morgan PT White MA. Night eating in obese treatment-seeking Hispanic patients with and without binge eating disorder. *Int J Eat Disord.* (2012) 45:787–91. 10.1002/eat.22011 22407481 PMC3378792

[25] Downes MJ Brennan ML Williams HC Dean RS. Development of a critical appraisal tool to assess the quality of cross-sectional studies (AXIS). *BMJ Open.* (2016) 6:e011458. 10.1136/bmjopen-2016-011458 27932337 PMC5168618

[26] Sterne JA Hernan MA Reeves BC Savovic J Berkman ND Viswanathan M ROBINS-I: a tool for assessing risk of bias in non-randomised studies of interventions. *BMJ.* (2016) 355:i4919. 10.1136/bmj.i4919 27733354 PMC5062054

[27] Sterne JAC Savovic J Page MJ Elbers RG Blencowe NS Boutron I RoB 2: a revised tool for assessing risk of bias in randomised trials. *BMJ.* (2019) 366:l4898. 10.1136/bmj.l4898 31462531

[28] Weltzin TE Hsu LK Pollice C Kaye WH. Feeding patterns in bulimia nervosa. *Biol Psychiatry.* (1991) 30:1093–110. 10.1016/0006-3223(91)90180-T 1777527

[29] Masheb RM Grilo CM. Eating patterns and breakfast consumption in obese patients with binge eating disorder. *Behav Res Ther.* (2006) 44:1545–53. 10.1016/j.brat.2005.10.013 16376851

[30] Masheb RM Grilo CM White MA. An examination of eating patterns in community women with bulimia nervosa and binge eating disorder. *Int J Eat Disord.* (2011) 44:618–24. 10.1002/eat.20853 21997425 PMC3646558

[31] Harvey K Rosselli F Wilson GT Debar LL Striegel-Moore RH. Eating patterns in patients with spectrum binge-eating disorder. *Int J Eat Disord.* (2011) 44:447–51. 10.1002/eat.20839 21661003 PMC3113648

[32] Leblanc V Provencher V Begin C Gagnon-Girouard MP Corneau L Tremblay A Associations between eating patterns, dietary intakes and eating behaviors in premenopausal overweight women. *Eat Behav.* (2012) 13:162–5. 10.1016/j.eatbeh.2011.12.002 22365804

[33] Shah N Passi V Bryson S Agras WS. Patterns of eating and abstinence in women treated for bulimia nervosa. *Int J Eat Disord.* (2005) 38:330–4. 10.1002/eat.20204 16231339

[34] Ellison JM Simonich HK Wonderlich SA Crosby RD Cao L Mitchell JE Meal patterning in the treatment of bulimia nervosa. *Eat Behav.* (2016) 20:39–42. 10.1016/j.eatbeh.2015.11.008 26630618 PMC4701380

[35] Cachelin FM Thomas C Vela A Gil-Rivas V. Associations between meal patterns, binge eating, and weight for Latinas. *Int J Eat Disord.* (2017) 50:32–9. 10.1002/eat.22580 27436488 PMC5191965

[36] Mitchell JE Hatsukami D Eckert ED Pyle RL. Characteristics of 275 patients with bulimia. *Am J Psychiatry.* (1985) 142:482–5. 10.1176/ajp.142.4.482 3856401

[37] Waters A Hill A Waller G. Internal and external antecedents of binge eating episodes in a group of women with bulimia nervosa. *Int J Eat Disord.* (2001) 29:17–22. 10.1002/1098-108X(200101)29:1<17::AID-EAT3>3.0.CO;2-R 11135328

[38] Ferrer-Garcia M Gutierrez-Maldonado J Pla J Riva G Andreu-Gracia A Dakanalis A Development of a VR application for binge eating treatment: identification of contexts and cues related to bingeing behavior in Spanish Italian patients. *Stud Health Technol Inform.* (2014) 199:71–5. 24875693

[39] Pla-Sanjuanelo J Ferrer-Garcia M Gutierrez-Maldonado J Riva G Andreu-Gracia A Dakanalis A Identifying specific cues and contexts related to bingeing behavior for the development of effective virtual environments. *Appetite.* (2015) 87:81–9. 10.1016/j.appet.2014.12.098 25526828

[40] Blouin A Blouin J Aubin P Carter J Goldstein C Boyer H Seasonal patterns of bulimia nervosa. *Am J Psychiatry.* (1992) 149:73–81. 10.1176/ajp.149.1.73 1728189

[41] Elran-Barak R Accurso EC Goldschmidt AB Sztainer M Byrne C Le Grange D. Eating patterns in youth with restricting and binge eating/purging type anorexia nervosa. *Int J Eat Disord.* (2014) 47:878–83. 10.1002/eat.22289 24777645 PMC4337799

[42] Carnell S Grillot C Ungredda T Ellis S Mehta N Holst J Morning and afternoon appetite and gut hormone responses to meal and stress challenges in obese individuals with and without binge eating disorder. *Int J Obes.* (2018) 42:841–9. 10.1038/ijo.2017.307 29235554

[43] Taylor AE Hubbard J Anderson EJ. Impact of binge eating on metabolic and leptin dynamics in normal young women. *J Clin Endocrinol Metab.* (1999) 84:428–34. 10.1210/jc.84.2.428 10022396

[44] Tzischinsky O Latzer Y Epstein R Tov N. Sleep-wake cycles in women with binge eating disorder. *Int J Eat Disord.* (2000) 27:43–8. 10.1002/(SICI)1098-108X(200001)27:1<43::AID-EAT5>3.0.CO;2-Z 10590448

[45] Tzischinsky O Latzer Y. Sleep-wake cycles in obese children with and without binge-eating episodes. *J Paediatr Child Health.* (2006) 42:688–93. 10.1111/j.1440-1754.2006.00952.x 17044895

[46] Galasso L Montaruli A Mule A Castelli L Bruno E Caumo A The multidisciplinary therapy in binge eating disorder is able to influence the interdaily stability and sleep quality? *Chronobiol Int.* (2019) 36:1311–5. 10.1080/07420528.2019.1650059 31401875

[47] Mason TB Engwall A Mead MP Irish LA. Sleep and eating disorders among adults enrolled in a commercial weight loss program: associations with self-report and objective sleep measures. *Eat Weight Disord.* (2019) 24:307–12. 10.1007/s40519-019-00664-1 30852800

[48] Roveda E Montaruli A Galasso L Pesenti C Bruno E Pasanisi P Rest-activity circadian rhythm and sleep quality in patients with binge eating disorder. *Chronobiol Int.* (2018) 35:198–207. 10.1080/07420528.2017.1392549 29144185

[49] Harb A Levandovski R Oliveira C Caumo W Allison KC Stunkard A Night eating patterns and chronotypes: a correlation with binge eating behaviors. *Psychiatry Res.* (2012) 200:489–93. 10.1016/j.psychres.2012.07.004 22906954

[50] Vogel SW Bijlenga D Tanke M Bron TI van der Heijden KB Swaab H Circadian rhythm disruption as a link between attention-deficit/hyperactivity disorder and obesity? *J Psychosom Res.* (2015) 79:443–50. 10.1016/j.jpsychores.2015.10.002 26526321

[51] Romo-Nava F Blom TJ Cuellar-Barboza AB Winham SJ Colby CL Nunez NA Evening chronotype as a discrete clinical subphenotype in bipolar disorder. *J Affect Disord.* (2020) 266:556–62. 10.1016/j.jad.2020.01.151 32056926

[52] Monteleone P Tortorella A Docimo L Maldonato MN Canestrelli B De Luca L Investigation of 3111T/C polymorphism of the CLOCK gene in obese individuals with or without binge eating disorder: association with higher body mass index. *Neurosci Lett.* (2008) 435:30–3. 10.1016/j.neulet.2008.02.003 18314271

[53] Stunkard AJ Grace WJ Wolff HG. The night-eating syndrome; a pattern of food intake among certain obese patients. *Am J Med.* (1955) 19:78–86. 10.1016/0002-9343(55)90276-X 14388031

[54] Allison KC Lundgren JD O’Reardon JP Geliebter A Gluck ME Vinai P Proposed diagnostic criteria for night eating syndrome. *Int J Eat Disord.* (2010) 43:241–7. 10.1002/eat.20693 19378289 PMC4531092

[55] Goel N Stunkard AJ Rogers NL Van Dongen HP Allison KC O’Reardon JP Circadian rhythm profiles in women with night eating syndrome. *J Biol Rhythms.* (2009) 24:85–94. 10.1177/0748730408328914 19150931 PMC3564642

[56] Schenck CH Hurwitz TD O’Connor KA Mahowald MW. Additional categories of sleep-related eating disorders and the current status of treatment. *Sleep.* (1993) 16:457–66. 8104356

[57] Greeno CG Wing RR Marcus MD. Nocturnal eating in binge eating disorder and matched-weight controls. *Int J Eat Disord.* (1995) 18:343–9. 10.1002/1098-108X(199512)18:4<343::AID-EAT2260180407>3.0.CO;2-P 8580920

[58] Allison KC Grilo CM Masheb RM Stunkard AJ. Binge eating disorder and night eating syndrome: a comparative study of disordered eating. *J Consult Clin Psychol.* (2005) 73:1107–15. 10.1037/0022-006X.73.6.1107 16392984

[59] Rand CS Macgregor AM Stunkard AJ. The night eating syndrome in the general population and among postoperative obesity surgery patients. *Int J Eat Disord.* (1997) 22:65–9. 10.1002/(SICI)1098-108X(199707)22:1<65::AID-EAT8>3.0.CO;2-0 9140737

[60] Adami GF Meneghelli A Scopinaro N. Night eating and binge eating disorder in obese patients. *Int J Eat Disord.* (1999) 25:335–8. 10.1002/(SICI)1098-108X(199904)25:3<335::AID-EAT12>3.0.CO;2-1 10191999

[61] Grilo CM Masheb RM. Night-time eating in men and women with binge eating disorder. *Behav Res Ther.* (2004) 42:397–407. 10.1016/S0005-7967(03)00148-7 14998734

[62] Allison KC Wadden TA Sarwer DB Fabricatore AN Crerand CE Gibbons LM Night eating syndrome and binge eating disorder among persons seeking bariatric surgery: prevalence and related features. *Obesity.* (2006) 14 (Suppl. 2):77S–82S. 10.1038/oby.2006.286 16648598

[63] Striegel-Moore RH Dohm FA Hook JM Schreiber GB Crawford PB Daniels SR. Night eating syndrome in young adult women: prevalence and correlates. *Int J Eat Disord.* (2005) 37:200–6. 10.1002/eat.20128 15822078

[64] Tholin S Lindroos A Tynelius P Akerstedt T Stunkard AJ Bulik CM Prevalence of night eating in obese and nonobese twins. *Obesity.* (2009) 17:1050–5. 10.1038/oby.2008.676 19396084 PMC2923060

[65] Root TL Thornton LM Lindroos AK Stunkard AJ Lichtenstein P Pedersen NL Shared and unique genetic and environmental influences on binge eating and night eating: a Swedish twin study. *Eat Behav.* (2010) 11:92–8. 10.1016/j.eatbeh.2009.10.004 20188292 PMC2830904

[66] Runfola CD Allison KC Hardy KK Lock J Peebles R. Prevalence and clinical significance of night eating syndrome in university students. *J Adolesc Health.* (2014) 55:41–8. 10.1016/j.jadohealth.2013.11.012 24485551 PMC4065810

[67] Napolitano MA Head S Babyak MA Blumenthal JA. Binge eating disorder and night eating syndrome: psychological and behavioral characteristics. *Int J Eat Disord.* (2001) 30:193–203. 10.1002/eat.1072 11449453

[68] Sassaroli S Ruggiero GM Vinai P Cardetti S Carpegna G Ferrato N Daily and nightly anxiety among patients affected by night eating syndrome and binge eating disorder. *Eat Disord.* (2009) 17:140–5. 10.1080/10640260802714597 19242843

[69] Meule A Allison KC Platte P. Emotional eating moderates the relationship of night eating with binge eating and body mass. *Eur Eat Disord Rev.* (2014) 22:147–51. 10.1002/erv.2272 24293184

[70] Braun DL Sunday SR Fornari VM Halmi KA. Bright light therapy decreases winter binge frequency in women with bulimia nervosa: a double-blind, placebo-controlled study. *Compr Psychiatry.* (1999) 40:442–8. 10.1016/S0010-440X(99)90088-3 10579376

[71] Lam RW Goldner EM Solyom L Remick RA. A controlled study of light therapy for bulimia nervosa. *Am J Psychiatry.* (1994) 151:744–50. 10.1176/ajp.151.5.744 8166318

[72] Lam RW Lee SK Tam EM Grewal A Yatham LN. An open trial of light therapy for women with seasonal affective disorder and comorbid bulimia nervosa. *J Clin Psychiatry.* (2001) 62:164–8. 10.4088/JCP.v62n0305 11305701

[73] De Young KP Thiel A Goodman EL Murtha-Berg E Johnson NK. A preliminary mechanistic test of the effects of light therapy in bulimia nervosa. *Adv Eat Disord.* (2016) 4:237–49. 10.1080/21662630.2016.1198980

[74] Blouin AG Blouin JH Iversen H Carter J Goldstein C Goldfield G Light therapy in bulimia nervosa: a double-blind, placebo-controlled study. *Psychiatry Res.* (1996) 60:1–9. 10.1016/0165-1781(95)02532-4 8852863

[75] Leung M Tranmer J Hung E Korsiak J Day AG Aronson KJ. Shift work, chronotype, and melatonin patterns among female hospital employees on day and night Shifts. *Cancer Epidemiol Biomarkers Prev.* (2016) 25:830–8. 10.1158/1055-9965.EPI-15-1178 26941366

[76] Jung H Dan H Pang Y Kim B Jeong H Lee JE Association between dietary habits, shift work, and the metabolic syndrome: the Korea Nurses’ health study. *Int J Environ Res Public Health.* (2020) 17:7697. 10.3390/ijerph17207697 33096883 PMC7589731

[77] Razavi P Devore EE Bajaj A Lockley SW Figueiro MG Ricchiuti V Shift work, chronotype, and melatonin rhythm in nurses. *Cancer Epidemiol Biomarkers Prev.* (2019) 28:1177–86. 10.1158/1055-9965.EPI-18-1018 31142495 PMC6750706

[78] Kim O Jung H. Prediction model for abnormal eating behaviour among hospital nurses: a structural equation modelling approach. *Int J Nurs Pract.* (2021) 27:e13006. 10.1111/ijn.13006 34363295

[79] Kosmadopoulos A Kervezee L Boudreau P Gonzales-Aste F Vujovic N Scheer F Effects of shift work on the eating behavior of police officers on patrol. *Nutrients.* (2020) 12:999. 10.3390/nu12040999 32260404 PMC7230712

[80] Shaw E Dorrian J Coates AM Leung GKW Davis R Rosbotham E Temporal pattern of eating in night shift workers. *Chronobiol Int.* (2019) 36:1613–25. 10.1080/07420528.2019.1660358 31495232

[81] Weltzin TE McConaha C McKee M Hsu LK Perel J Kaye WH. Circadian patterns of cortisol, prolactin, and growth hormonal secretion during bingeing and vomiting in normal weight bulimic patients. *Biol Psychiatry.* (1991) 30:37–48. 10.1016/0006-3223(91)90068-W 1892960

[82] Bulik CM Coleman JRI Hardaway JA Breithaupt L Watson HJ Bryant CD Genetics and neurobiology of eating disorders. *Nat Neurosci.* (2022) 25:543–54. 10.1038/s41593-022-01071-z 35524137 PMC9744360

[83] Facer-Childs ER Middleton B Skene DJ Bagshaw AP. Resetting the late timing of ‘night owls’ has a positive impact on mental health and performance. *Sleep Med.* (2019) 60:236–47. 10.1016/j.sleep.2019.05.001 31202686

[84] Burke TM Markwald RR Chinoy ED Snider JA Bessman SC Jung CM Combination of light and melatonin time cues for phase advancing the human circadian clock. *Sleep.* (2013) 36:1617–24. 10.5665/sleep.3110 24179293 PMC3792377

[85] Xiao Q Garaulet M Scheer F. Meal timing and obesity: interactions with macronutrient intake and chronotype. *Int J Obes.* (2019) 43:1701–11. 10.1038/s41366-018-0284-x 30705391 PMC6669101

[86] Wehrens SMT Christou S Isherwood C Middleton B Gibbs MA Archer SN Meal timing regulates the human circadian system. *Curr Biol.* (2017) 27:1768–75.e3. 10.1016/j.cub.2017.04.059 28578930 PMC5483233

[87] Plano SA Soneira S Tortello C Golombek DA. Is the binge-eating disorder a circadian disorder? *Front Nutr.* (2022) 9:964491. 10.3389/fnut.2022.964491 35938096 PMC9352861

[88] Osnaya-Ramirez RI Palma-Gomez M Escobar C. Binge eating for sucrose is time of day dependent and independent of food restriction: effects on mesolimbic structures. *Behav Neurosci.* (2020) 134:267–81. 10.1037/bne0000364 32150421

[89] Romo-Nava F Buijs FN Valdes-Tovar M Benitez-King G Basualdo M Perusquia M Olanzapine-induced early cardiovascular effects are mediated by the biological clock and prevented by melatonin. *J Pineal Res.* (2017) 62:12402. 10.1111/jpi.12402 28226198

[90] Gebhardt S Haberhausen M Krieg JC Remschmidt H Heinzel-Gutenbrunner M Hebebrand J Clozapine/olanzapine-induced recurrence or deterioration of binge eating-related eating disorders. *J Neural Transm.* (2007) 114:1091–5. 10.1007/s00702-007-0663-2 17372672

[91] McElroy SL Guerdjikova AI Mori N Romo-Nava F. Progress in developing pharmacologic agents to treat bulimia nervosa. *CNS Drugs.* (2019) 33:31–46. 10.1007/s40263-018-0594-5 30523523

