# Supplementary material for: A matter of time: A systematic scoping review on a potential role of the circadian system in binge eating behavior
Source: Front Nutr. 2022 Sep 8;9:978412. doi: 10.3389/fnut.2022.978412 (PMC9493346; doi:10.3389/fnut.2022.978412)
Supplement: Supplementary file 3 [file Table_3.DOCX]

|  |  |  |  |  |  |  |  |  |  |  |  |  |  |
| --- | --- | --- | --- | --- | --- | --- | --- | --- | --- | --- | --- | --- | --- |
|  | **Supplementary table 3. Assessment of circadian-related measures among individuals with BE behavior critical appraisal of risk of bias (Axis criteria)** | | | | | | | | | | | | |
|  |  | **Carnell S, 2018** | **Galasso L, 2019** | **Harb, 2012** | **Mason TB, 2019** | **Monteleone, 2008** | **Romo-Nava F, 2020** | **Roveda E, 2018** | **Taylor AE, 1999** | **Tzichinsky, 2000** | **Tzinchinsky, 2006** | **Weltzin TE, 1991** | **Vogel S, 2015** |
|  | **Introduction** |  |  |  |  |  |  |  |  |  |  |  |  |
| 1 | Were the aims/objectives of thestudy clear? | Y | Y | Y | Y | Y | Y | Y | Y | Y | Y | Y | Y |
|  | **Methods** |  |  |  |  |  |  |  |  |  |  |  |  |
| 2 | Was the study design appropriate for the  stated aim(s)? | Y | Y | Y | Y | Y | Y | Y | Y | Y | Y | Y | Y |
| 3 | Was the sample size justified? | Y | N | Y | Y | Y | Y | N | N | N | N | N | Y |
| 4 | Was the target/reference population clearly defined? (Is it clear who the research was about?) | Y | Y | Y | Y | Y | Y | Y | Y | Y | Y | Y | Y |
| 5 | Was the sample frame taken from an appropriate population base so that it closely represented the target/reference population under investigation? | Y | Y | Y | Y | Y | Y | Y | Y | Y | Y | Y | Y |
| 6 | Was the selection process likely to select subjects/participants that were representative of the target/reference population under investigation? | Y | Y | Y | Y | Y | Y | Y | Y | Y | Y | Y | Y |
| 7 | Were measures undertaken to address and categorise non-responders? | N | N | N | Y | N | N | N | N | N | N | N | N |
| 8 | Were the risk factor and outcome variables measured appropriate to the aims of the study? | Y | Y | Y | Y | Y | Y | Y | Y | Y | Y | Y | Y |
| 9 | Were the risk factor and outcome variables measured correctly using instruments/measurements that had been trialled, piloted or published previously? | Y | Y | Y | Y | Y | Y | Y | Y | Y | Y | Y | Y |
| 10 | Is it clear what was used to determinef statistical significance and/or precision estimates? (e.g. p-values, confidence intervals) | Y | Y | Y | Y | Y | Y | Y | Y | Y | Y | Y | Y |
| 11 | Were the methods (including statistical methods) sufficiently described to enable them to be repeated? | Y | Y | Y | Y | Y | Y | Y | Y | Y | Y | Y | Y |
|  | **Results** |  |  |  |  |  |  |  |  |  |  |  |  |
| 12 | Were the basic data adequately described? | Y | Y | Y | Y | Y | Y | Y | Y | Y | Y | Y | Y |
| 13 | Does the response rate raise concerns about non-response bias? | ? | ? | ? | N | ? | ? | ? | ? | ? | ? | ? | ? |
| 14 | If appropriate, was information about  non-responders described? | ? | ? | ? | Y | ? | ? | ? | ? | ? | ? | ? | ? |
| 15 | Were the results internally consistent? | Y | Y | Y | Y | Y | Y | Y | Y | Y | Y | Y | Y |
| 16 | Were the results presented for all the  analyses described in the methods? | Y | Y | Y | Y | Y | Y | Y | Y | Y | Y | Y | Y |
|  | **Discussion** |  |  |  |  |  |  |  |  |  |  |  |  |
| 17 | Were the authors' discussions and  conclusions justified by the results? | Y | Y | Y | Y | Y | Y | Y | Y | Y | Y | Y | Y |
| 18 | Were the limitations of the study discussed? | Y | N | Y | Y | Y | Y | Y | Y | N | Y | Y | Y |
|  | **Other** |  |  |  |  |  |  |  |  |  |  |  |  |
| 19 | Were there any funding sources or  conflicts of interest that may affect the authors’ interpretation of the results? | N | N | ? | N | ? | N | N | ? | ? | ? | ? | N |
| 20 | Was ethical approval or consent of participants attained? | Y | ? | Y | Y | Y | Y | Y | Y | Y | Y | ? | Y |
|  |  |  |  |  |  |  |  |  |  |  |  |  |  |
|  | Y= Yes |  |  |  |  |  |  |  |  |  |  |  |  |
|  | N= No |  |  |  |  |  |  |  |  |  |  |  |  |
|  | ?= Unknown |  |  |  |  |  |  |  |  |  |  |  |  |
