# Supplementary material for: A matter of time: A systematic scoping review on a potential role of the circadian system in binge eating behavior
Source: Front Nutr. 2022 Sep 8;9:978412. doi: 10.3389/fnut.2022.978412 (PMC9493346; doi:10.3389/fnut.2022.978412)
Supplement: Supplementary file 4 [file Table_4.DOCX]

|  | **Supplementary table 4. Studies on the overlap of BE and night eating behavior critical appraisal of risk of bias (AXIS Criteria).** | | | | | | | | | | | | | | | | | | | |
| --- | --- | --- | --- | --- | --- | --- | --- | --- | --- | --- | --- | --- | --- | --- | --- | --- | --- | --- | --- | --- |
|  |  | **Adami GF, 1999** | **Allison KC, 2005** | **Allison KC,2006** | **Colles SL,2007** | **de Zwaan M, 2006** | **Greeno CG, 1995** | **Grilo CM,2004** | **Grilo CM,2012** | **Harb A,2012** | **Latzer Y, 2020** | **Meule A,2014** | **Napolitano MA, 2001** | **Rand CS, 1997** | **Root TL, 2010** | **Runfola CD, 2014** | **Sassaroli S,2009** | **Schenck CH, 1993** | **Striegel-Moore RH,2010** | **Tholin S, 2009** |
|  | **Introduction** |  |  |  |  |  |  |  |  |  |  |  |  |  |  |  |  |  |  |  |
| 1 | Were the aims/objectives of thestudy clear? | Y | Y | Y | Y | Y | Y | Y | Y | Y | Y | Y | Y | Y | Y | Y | Y | Y | Y | Y |
|  | **Methods** |  |  |  |  |  |  |  |  |  |  |  |  |  |  |  |  |  |  |  |
| 2 | Was the study design appropriate for the stated aim(s)? | Y | Y | Y | Y | Y | Y | Y | Y | Y | Y | Y | Y | Y | Y | Y | Y | Y | Y | Y |
| 3 | Was the sample size justified? | N | Y | Y | Y | Y | N | Y | Y | Y | Y | Y | Y | N | Y | Y | N | N | Y | Y |
| 4 | Was the target/reference population clearly defined? (Is it clear who the research was about?) | Y | Y | Y | Y | Y | Y | Y | Y | Y | Y | Y | Y | Y | Y | Y | Y | Y | Y | Y |
| 5 | Was the sample frame taken from an  appropriate population base so that it closely represented the target/reference population under investigation? | Y | Y | Y | Y | Y | Y | Y | Y | Y | Y | Y | Y | Y | Y | Y | Y | Y | Y | Y |
| 6 | Was the selection process likely to  select subjects/participants that were representative of the target/reference population under investigation? | Y | Y | Y | Y | Y | Y | Y | Y | Y | Y | Y | Y | Y | Y | Y | Y | Y | Y | Y |
| 7 | Were measures undertaken to address  and categorise non-responders? | N | Y | Y | Y | Y | N | N | N | N | Y | N | Y | N | Y | Y | N | N | N | Y |
| 8 | Were the risk factor and outcome  variables measured appropriate to the aims of the study? | ? | Y | Y | Y | Y | Y | Y | Y | Y | Y | Y | Y | Y | Y | Y | Y | Y | Y | Y |
| 9 | Were the risk factor and outcome variables measured correctly using instruments/measurements that had been trialled, piloted or published previously? | N | Y | Y | Y | Y | Y | Y | Y | Y | Y | Y | Y | Y | Y | Y | Y | Y | Y | Y |
| 10 | Is it clear what was used to determined  statistical significance and/or precision estimates? (e.g. p-values, confidence intervals) | N | Y | Y | Y | Y | N | Y | Y | Y | Y | Y | Y | Y | Y | Y | Y | Y | Y | Y |
| 11 | Were the methods (including statistical methods) sufficiently described to enable them to be repeated? | N | Y | Y | Y | Y | N | Y | Y | Y | Y | Y | Y | Y | Y | Y | Y | Y | Y | Y |
|  | **Results** |  |  |  |  |  |  |  |  |  |  |  |  |  |  |  |  |  |  |  |
| 12 | Were the basic data adequately described? | N | Y | Y | Y | Y | N | Y | Y | Y | Y | Y | Y | N | Y | Y | Y | Y | Y | Y |
| 13 | Does the response rate raise concerns about non-response bias? | ? | N | N | N | N | ? | ? | ? | ? | N | ? | N | ? | N | N | N | ? | ? | N |
| 14 | If appropriate, was information about  non-responders described? | ? | Y | Y | Y | Y | ? | ? | ? | ? | Y | ? | Y | ? | Y | Y | Y | ? | ? | Y |
| 15 | Were the results internally consistent? | Y | Y | Y | Y | Y | Y | Y | Y | Y | Y | Y | Y | Y | Y | Y | Y | Y | Y | Y |
| 16 | Were the results presented for all the  analyses described in the methods? | Y | Y | Y | Y | Y | N | Y | Y | Y | Y | Y | Y | Y | Y | Y | Y | Y | Y | Y |
|  | **Discussion** |  |  |  |  |  |  |  |  |  |  |  |  |  |  |  |  |  |  |  |
| 17 | Were the authors' discussions and  conclusions justified by the results? | Y | Y | Y | Y | Y | N | Y | Y | Y | Y | Y | Y | N | Y | Y | Y | Y | Y | Y |
| 18 | Were the limitations of the study discussed? | N | Y | N | Y | Y | N | Y | Y | Y | Y | Y | Y | N | Y | Y | N | N | Y | Y |
|  | **Other** |  |  |  |  |  |  |  |  |  |  |  |  |  |  |  |  |  |  |  |
| 19 | Were there any funding sources or conflicts  of interest that may affect the authors’ interpretation of the results? | ? | ? | ? | ? | ? | ? | ? | N | ? | Y | ? | ? | ? | Y | Y | ? | ? | ? | Y |
| 20 | Was ethical approval or consent of participants  attained? | ? | Y | Y | Y | Y | ? | Y | Y | Y | Y | Y | ? | ? | Y | Y | Y | ? | ? | Y |
|  |  |  |  |  |  |  |  |  |  |  |  |  |  |  |  |  |  |  |  |  |
|  | Y= Yes |  |  |  |  |  |  |  |  |  |  |  |  |  |  |  |  |  |  |  |
|  | N= No |  |  |  |  |  |  |  |  |  |  |  |  |  |  |  |  |  |  |  |
|  | ?= Unknown |  |  |  |  |  |  |  |  |  |  |  |  |  |  |  |  |  |  |  |
