# Supplementary figures and images for: A matter of time: A systematic scoping review on a potential role of the circadian system in binge eating behavior
Source: Front Nutr. 2022 Sep 8;9:978412. doi: 10.3389/fnut.2022.978412 (PMC9493346; doi:10.3389/fnut.2022.978412)

**Supplementary figure 1.** Risk of bias for non-randomized interventional studies

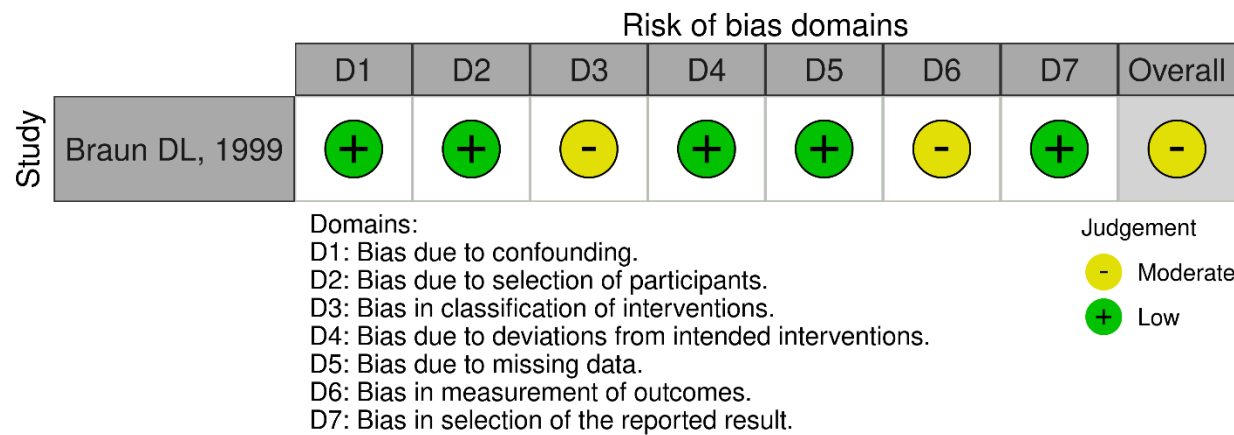

Supplement: Supplementary file 5 [file Image_1.pdf]
