# Supplementary material for: A matter of time: A systematic scoping review on a potential role of the circadian system in binge eating behavior
Source: Front Nutr. 2022 Sep 8;9:978412. doi: 10.3389/fnut.2022.978412 (PMC9493346; doi:10.3389/fnut.2022.978412)

Supplementary figure 2. Risk of bias for randomized interventional studies.

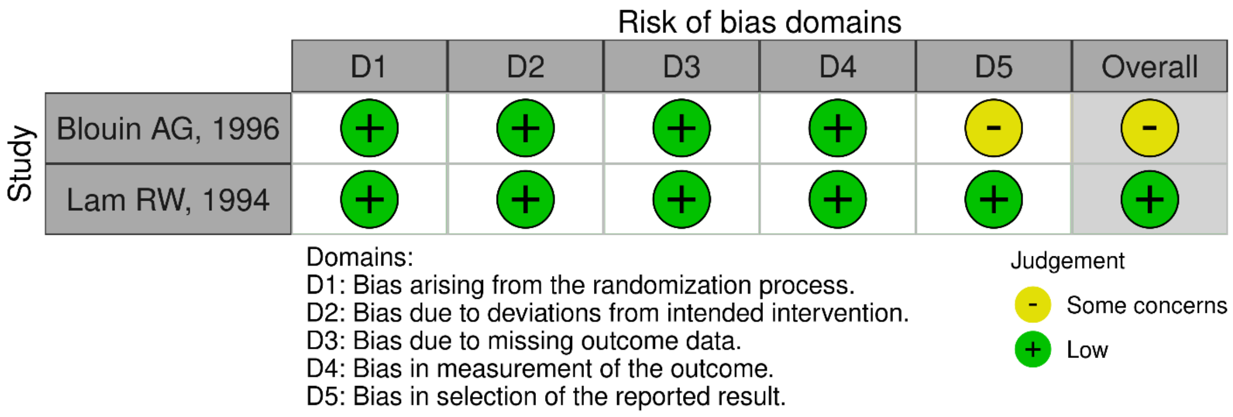

Supplement: Supplementary file 6 [file Image_2.pdf]
